# From 19th-century mysteries to modern insights: untangling *Aplectana membranosa* from Brazilian anurans

**DOI:** 10.1017/S0031182025000204

**Published:** 2025-03

**Authors:** Ana Nunes Santos, Evelyn Lebrego Cardoso, Lorena Freitas Souza Tavares-Costa, Rayline Thaimenne Alves Figueredo, Gabriel Lima Rebêlo, Maria Isabel Müller, Edna P. Alcantara, Edson A. Adriano, Drausio Honorio Morais, Simone Mousinho Freire, Jeannie Nascimento Dos Santos, Francisco Tiago de Vasconcelos Melo

**Affiliations:** 1 Laboratório de Biologia Celular e Helmintologia ‘Profa Dra Reinalda Marisa Lanfredi’ Instituto de Ciências Biológicas, Universidade Federal do Pará, Belém, PA, Brasil; 2 Departamento de Biologia Animal, Universidade Estadual de Campinas-UNICAMP, Campinas, SP, Brasil; 3 Instituto de Ciências Ambientais, Químicas e Farmacêuticas, Universidade Federal de São Paulo, SP, Brasil; 4 Oregon State University, Department of Microbiology, Corvallis, OR, USA; 5 Instituto de Ciências Agrárias, Universidade Federal de Uberlândia (UFU), Monte Carmelo, MG, Brasil; 6 Departamento de Biologia, Laboratório de Zoologia e Biologia Parasitária, Universidade Estadual do Piauí, Teresina, PI, Brasil

**Keywords:** *Aplectana membranosa*, integrative taxonomy, metric variation, population divergence

## Abstract

*Aplectana membranosa* is a cosmocercid nematode that shows affinity with various amphibian and reptile hosts, being considered a generalist species. To date, no studies have investigated the influence of host and locality in the morphological variation of this species. Thus, we analysed morphological and morphometric characters of 260 specimens of *A. membranosa* collected from 9 host species and 7 different localities. To complement the metric studies, we conducted phylogenetic analyses using the ribosomal genes *28S* and internal transcript spacer 1 (*ITS1*) to determine the phylogenetic position of the species and its divergence. In the present study, it was possible to observe the cloacal papillae pattern of the species through scanning electron microscopy, and we found no morphological variation in the specimens of *A. membranosa* from various hosts in different localities in Brazil. The study showed low variation in all data. However, despite the low variation, we found that external environmental conditions, such as climate and latitude, influence its variation. Molecular analyses highlighted that the separation of Cosmocercidae members may be related to geographic distribution and population genetic divergence. Thus, the results illustrated in this study reiterate the importance of using integrative data to better elucidate the family’s taxonomic and evolutionary history.

## Graphical abstract

**Figure:**
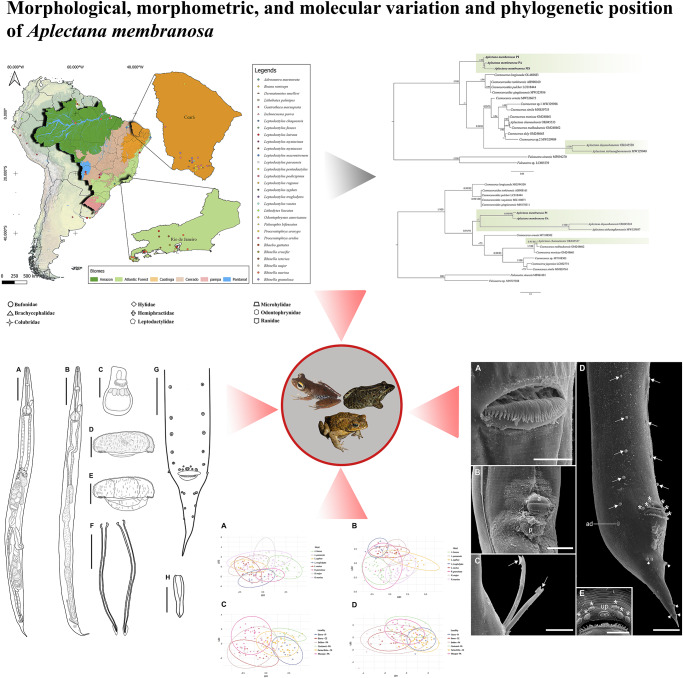

## Introduction

*Aplectana membranosa* (Schneider, 1866) Miranda, 1924 belongs to the family Cosmocercidae Travassos, 1925 and was originally described as *Leptodera membranosa* Schneider, 1866, which was found parasitizing a species of frog from Brazil (Schneider, 1866) and later reassigned to the genus *Aplectana* (Miranda, 1924). The original description of *A. membranosa* by Schneider (1866) is incomplete. The author did not clarify the set of characteristics for identifying the species, nor did he determine the host and type locality. Miranda (1924) redescribed the species and established some characteristics for the diagnosis of the taxon. However, it is still unclear whether the specimens analysed by the author are the same as those found by Schneider (1866).

This nematode is widely found to parasitize several species of hosts in the Neotropics (Lins et al., 2017; Cardoso et al., 2021; Chero et al., 2023). In Brazil, *A. membranosa* was found to parasitize 16 frog species of 6 different families, namely, Bufonidae, Brachycephalidae, Hylidae, Leptodactylidae, Microhylidae and Odontophrynidae, occurring in the states of Amazonas, Pará, Ceará, Mato Grosso do Sul, Rio de Janeiro and São Paulo (Gonçalves et al., 2002; Luque et al., 2005; Martins and Fabio, 2005; Alcantara et al., 2018; Silva et al., 2019; Cardoso et al., 2021; Mascarenhas et al., 2021; Sani et al., 2021; Vieira et al., 2021). Thus, *A. membranosa* is considered a generalist species (Teles et al., 2018; Gómez et al., 2020; Cardoso et al., 2021; Sampaio et al., 2022).

Various hosts can generate different selective pressures in a species, leading to morphological, morphometric and genetic differences (Mayr, 1963; Archie and Ezenwa, 2011; Losos, 2011; Vázquez-Prieto et al., 2015). For example, *Aplectana hylambatis* Baylis, 1927, *A. mancintoshi* (Velasquez, 1959) and *A. hamatospicula* (Walton, 1940) exhibit morphological and morphometric variation related to their hosts and localities (Vhora and Bolek, 2013; Ibraheem et al., 2017; González et al., 2019). These intraspecific variations may hinder the identification of taxa (Hoberg and Brooks, 2008; Araujo et al., 2015).

*Aplectana membranosa* is widely distributed in Brazil and Peru. No studies have presented molecular data or detailed its morphological and morphometric variation. Our study aimed to evaluate whether different host species and localities influence the morphology, morphometry and genetics of *A. membranosa*. For this purpose, we used parasites of 9 anuran species from 5 Brazilian states and determined the species’ phylogenetic position using the ribosomal genes *28S* and *ITS1*.

## Materials and methods

### Collection of hosts and parasites

We analysed 132 hosts distributed in 3 families, Bufonidae Gray, 1825; Leptodactylidae Werner, 1896 (1838); and Hylidae Rafinesque, 1815, which include 9 species (10 specimens per species per locality), from 7 localities in 5 Brazilian states: Amapá (AP), Ceará (CE), Pará (PA), Piauí (PI) and Mato Grosso do Sul (MS) (Table 1).

**Table 1.** Number of host species collected and localities of *A. membranosa* obtained in this study

Localities	Host family/species	Number of hosts collected
Macapá, Amapá	**Bufonidae**	
	*Rhinella major*	10
	*Rhinella marina*	10
	**Leptodactylidae**	
	*Leptodactylus fuscus*	10
Belém, Pará (Universidade Federal do Pará)^a^	**Bufonidae**	
	*Rhinella marina*^b^	10
	**Leptodactylidae**	
	*Leptodactylus fuscus*	10
	*Leptodactylus paraensis*	10
FLONA Caxiuanã, Pará	**Bufonidae**	
	*Rhinella marina*	10
Barras, Piauí^a^	**Leptodactylidae**	
	*Leptodactylus vastus*	10
	**Hylidae**	
	*Scinax ruber*^a^	1
Barro, Ceará	**Leptodactylidae**	
	*Leptodactylus fuscus*	10
Farias de Brito, Ceará	**Bufonidae**	
	*Rhinella granulosa*	10
	**Leptodactylidae**	
	*Leptodactylus troglodytes*	10
	*Leptodactylus syphax*	10
	*Leptodactylus vastus*	10
Brasilândia, Mato Grosso do Sul^a^	**Leptodactylidae**	
	*Leptodactylus latrans*^a^	1
		132

a Host species of specimens of *A. membranosa* used only for molecular analysis.

b Host species of specimens of *A. membranosa* used for molecular analysis, morphological and morphometrical analysis.

The samples of *A. membranosa* for molecular analyses were collected from *Leptodactylus latrans* (Steffen, 1815) from the state of Mato Grosso do Sul, *Scinax ruber* (Laurenti, 1768) from the state of Piauí and *Rhinella marina* (Linnaeus, 1758) from the state of Pará. *A. membranosa*, a parasite of *L. latrans* from Mato Grosso do Sul and a parasite of *S. ruber* from Piauí were only analysed for molecular characterization, as we did not adequate size for morphological and morphometric analyses, both in terms of the number of hosts and the number of parasites.

The hosts were transported to the laboratory, euthanized with 2% lidocaine, weighed and necropsied. The internal organs were removed, separated in Petri dishes containing saline solution (0.9% NaCl), dissected and examined for helminths under a Leica EZ4 stereomicroscope (Leica Microsystems, Wetzlar, Germany). The nematodes were washed in saline solution, killed with 70% alcohol, heated to 60 °C and preserved in the same solution at room temperature.

### Morphological and morphometric analysis of A. membranosa

We analysed 260 specimens (130 female and 130 male) of *A. membranosa*. Specimens were identified based on Schneider (1866) and Miranda (1924). For morphological and morphometric analysis, the nematodes were clarified in 20% Aman’s lactophenol, mounted on temporary slides and observed under an Olympus BX41 microscope (Olympus, Tokyo, Japan).

For scanning electron microscopy (SEM), the specimens were powder-fixed in OsO_4_, dehydrated in an ascending ethanol series, dried at the CO_2_ critical point, coated with palladium gold, mounted on metal supports and examined under a Vega3 microscope (TESCAN, Brno, Czech Republic) at the Laboratory of Structural Biology of the Federal University of Pará (UFPA).

The following male characters were considered for morphological analysis: number and arrangement of caudal papillae, shape of spicules and gubernaculum. For morphometry, 12 characters were taken into account: body length, body width at the oesophageal–gut junction, total oesophageal length, pharyngeal length, isthmus length, bulb length, bulb width, distance from the nerve ring to the anterior region, distance from the excretory pore to the anterior region, tail length (distance from the cloaca to the posterior end to the extremity) and length of the spicules and gubernaculum.

The terminology and pattern of the caudal papillae followed those proposed by González et al. (2019). Thus, we considered the number and distribution of pairs of *A. membranosa* papillae according to the following: 5 pairs of precloacal papillae in a row, a pair of adcloacal papillae (one papilla on each side of the cloaca); 3 pairs of papillae on the upper lip of the cloaca and 1 large simple papillae (1 unpaired:3 pairs); and 4 pairs of postcloacal papillae.

The morphological and morphometric characters considered for the females were the presence/absence and number of protuberances on the vulvar lip, body length, body width at the oesophageal–gut junction, total oesophageal length, pharyngeal length, isthmus length, bulb length, bulb width, distance from the nerve ring to the anterior region, distance from the excretory pore to the anterior region, distance from the vulva to the posterior region, length and width of the eggs and length of the tail.

All measurement values are given in micrometres unless otherwise indicated. For additional morphological comparisons, we examined specimens of *Aplectana membranosa* de Miranda (1924) deposited in the Helminthological Collection of the Instituto Oswaldo Cruz, Brazil (CHIOC), under the numbers CHIOC 1593 and CHIOC 1594.

### Data analyses

As proposed by González et al. (2019), we used principal component analysis (PCA) to estimate which morphological characters/variables were most relevant in the total variation explained by each component. Seventeen female variables and 16 male variables of *A. membranosa* were included in the PCA to evaluate the weight of each variable in the different components and their explained variance. The objective of PCA was to reduce the multivariate dataset into a smaller set of composite variables with limited loss of information (Mcgarigal et al., 2000).

To test the hypothesis that host species and locality influence the metric variables of males and females, we applied multivariate analysis of variance (MANOVA), which included the most relevant components indicated by PCA. For significant differences, 2-way ANOVA was performed for each variable, followed by Tukey’s post hoc test.

Additionally, we performed a linear discriminant functional analysis to determine which of the selected variables in females and males best discriminated nematodes isolated from different hosts and locations. Before the analyses, the variables were logarithmically transformed [ln(*x*)] in PAST 3.11 software (Hammer et al., 2001) to give them a normal distribution. The analyses were performed with the factoMineR (Lê et al., 2008), rstatix (Kassambara, 2023) and MASS (Venables and Ripley, 2002) packages in *R* 4.1.1.

### Molecular analysis and phylogenetic analysis

Specimens for molecular analysis were collected from *Rhinella marina, Scinax ruber* and *Leptodactylus latrans* from 3 Brazilian states: Pará, Piauí and Mato Grosso do Sul, respectively. Specimens from all study locations were used to attempt DNA extraction. However, amplification was not successful for all hosts and locations.

The nematodes selected for the molecular analysis were cut in the anterior and posterior regions to confirm the identity of each sample and deposited in the collection of Non-Arthropod invertebrates of the Museu Paraense Emílio Goeldi, Belém, PA. The middle portion of the nematodes was stored in 100% ethanol for further molecular characterization as proposed by Pleijel et al. (2008).

DNA was extracted from the midsection of the nematode body in 200 μL of 5% Chelex® molecular Biology Grade resin suspended in deionized water and 2 μL of proteinase K, according to the manufacturer’s protocol, and then incubated at 56 °C for 14 h. The material was boiled at 90 °C for 8 min and centrifuged at 14 000 rpm for 10 min. The regions of the partial ribosomal genes *28S* and *ITS1* were amplified by polymerase chain reaction (PCR) using specific primers and cycling conditions following the protocols established by Chen et al. (2018). The PCR products were visualized on a 1% agarose gel to determine the yield and size of the amplified fragments and were purified using a QIAquick PCR Purification Kit.

The sequencing of the amplicons followed the protocol of the Big Dye® Terminator v.3.1 Cycle Sequencing Kit, and the amplicons were sequenced in an ABI 3730 DNA analyser at the Center for Research on Stem Cells of the Human Genome of the Institute of Biosciences of Brazil, University of São Paulo, Brazil.

The sequences obtained were edited using Geneious 7.1.3 software (Kearse et al., 2012). Then, a search for similar sequences in the same genomic region was performed using the BLASTn algorithm in the National Center for Biotechnology Information (NCBI) database (http://www.ncbi.nml.nih.gov) (details of the sequences used in the present study are given in Table 2). We performed 2 alignments, 1 for each gene, using the standard parameters of Muscle software (Edgar, 2004) implemented in Geneious 7.1.3 software (Kearse et al., 2012). Alignments were cut off at the ends, and poorly aligned regions were excluded from the analyses (Tran et al., 2015).

**Table 2.** Representatives of Cosmocercidae used for phylogenetic analyses, information on host, locality and GenBank accession numbers

Species	Host species 28S/ITS1	Collection site	GenBank ID 28S	GenBank ID ITS 1	References
***Aplectana membranosa* (**Schneider, 1866; Miranda, 1924)	***Rhinella marina* (Linnaeus, 1758)**	**Brazil: Belém, Pará**	PQ569941	PQ592008	**Present study**
***A. membranosa***	***Scinax ruber* (Laurenti, 1768**)	**Brazil: Barras, Piauí**	PQ569939	PQ580741	**Present study**
***A. membranosa***	***Leptodactylus latrans* (Steffen, 1815)**	**Brazil: Brasilândia, Mato Grosso do Sul**	PQ569940	–	**Present stud**y
*A. chamaleonis* (Baylis, 1929)	*Hyperolius kivuensis* Ahl, 1931	Germany	OK045533	–	Chen et al. (2021a)
*A. chamaleonis*	*H. kivuensis*	Germany	–	OK045527OK045529	Chen et al. (2021a)
*A. dayaoshanensis* Chen et al. (2021)	*Sylvirana spinulosa* (Smith, 1923)	China: Dayao Mountain, Guangxi Province	OK045530	–	Chen et al. (2021b)
*A. dayaoshanensis*	*Polypedates megacephalus* (Hallowell, 1861)	China: Dayao Mountain, Guangxi Province	–	OK045526; OK045524	Chen et al. (2021b)
*A. xishuangbannaensis* Chen, Gu, Ni e Li, 2021	*P. megacephalus*	China: Yunnan Province	MW329040		Chen et al. (2021a)
*A. xishuangbannaensis*	*P. megacephalus*	China: Yunnan Province	–	MW329037	Chen et al. (2021a)
*Cosmocerca longicauda* (Linstow, 1885)	*Lissotriton vulgaris* (Linnaeus, 1758)	Germany	OL468683	–	Sinsch et al. (2017)
*C. longicauda*	*L. vulgaris*	Germany	–	MG594350	Sinsch et al. (2017)
*C. ornata* (Dujardin, 1845; Diesing, 1861)	*Sylvirana spinulosa* (Smith, 1923)	China: Guangxi Province	MW326675	–	Chen et al. (2020)
*C. ornata*	*S. spinulosa*	China: Guangxi Province	–	MT108302	Chen et al. (2021)
*Cosmocerca* sp.	*Duttaphrynus melanostictus* (Schneider, 1799)	China: Jinghong, Yunnan Province	–	MT108303	Chen et al. (2020)
*Cosmocerca* sp. 1	*D. melanostictus*	China: Yunnan Province	MW329988	–	Chen et al. (2021)
*C. símile* Chen, Zhang, Feng and Li, 2020	*Bufo gargarizans* Cantor, 1842	China: Yuyao, Zhejiang Province	MN839755	–	Chen et al. (2020)
*C. simile*	*B. gargarizans*	China: Yuyao, Zhejiang Province	–	MN839761	Chen et al. (2020)
*C. monicae* Harnoster et al., 2023	*Kassina senegalensis* (Duméril and Bibron, 1841)	South Africa	OM248661	–	Harnoster et al. (2022)
*C. monicae*	*K. senegalensis*	South Africa	–	OM248661	Harnoster et al. (2022)
*C. makhadoensis* Harnoster et al., 2023	*Phrynomantis bifasciatus* (Smith, 1847)	South Africa	OM248662	–	Harnoster et al. (2022)
*C. makhadoensis*	*P. bifasciatus*	South Africa	–	OM248662	Harnoster et al. (2022)
*C. daly* Harnoster et al., 2023	*Cacosternum boettgeri* (Boulenger, 1882)	South Africa	OM248663	–	Harnoster et al. (2022)
*C. japonica* Yamaguti, 1938	*Bufo formosus* Boulenger, 1883	Japan: Niigata, Sado	–	LC052774	Sato et al. (2015)
*Cosmocerca* sp. 2	*Hoplobatrachus rugulosus* (Wiegmann, 1834)	China: Guangxi Province	MW329989	–	Chen et al. (2021)
*Cosmocercoides tonkinensis* Tran et al., 2015	*Acanthosaura lepidogaster* (Cuvier, 1829)	Vietnam: Thanh Hoa Province, Pu Hu	AB908160	–	Tran et al. (2015)
*C. tonkinensis*	*A. lepidogaster*	Vietnam: Bac Giang Province, Tay Yen Tu	–	AB908161	Tran et al. (2015)
*C. pulcher* Wilkie, 1930	*Bufo japonicus* Temminck and Schlegel 1838	Japan: Oita	LC018444	–	Tran et al. (2015)
*C. pulcher*	*B. japonicus*	Japan: Oita	–	LC018444	Tran et al. (2015)
*C. qingtianensis* Chen et al., 2018	*B. gargarizans*	China: Henan Province	MW325956	–	Chen et al. (2021)
*C. qingtianensis*	*B. gargarizans*	China: Qingtian River scenic area, Jiaozuo, Henan Province	–	MH178311	Chen et al. (2018)
*C. wuyiensis*	*Amolops wuyiensis* (Liu and Hu, 1975)	China	–	MK110871	Liu et al. (2019)
*Falcaustra sinensis* Liu et al., 2011	*Indotestudo elongata* (Blyth, 1854)	China	MF094270	–	Li et al. (2018)
**Outgroup**					
*F. sinensis*	*I. elongata*	China	–	MF061681	Li et al. (2018)
*Falcaustra* sp.	*Andiras* sp.	Japan:Kyoto	LC605539	–	Tsuchida et al. (2023)
*Falcaustra* sp.	*Physignathus cocincinus* Cuvier, 1829	Vietnam	–	MN727388	Binh (2019)

Substitution saturation was evaluated on the aligned matrices, and the *Iss* index was estimated using the DAMBE 5 software package (Xia, 2013). The number of base substitutions between sequences per site was calculated. Standard error estimates were obtained using a bootstrap procedure with 1000 replicates. Genetic divergence was calculated for the matrix of each gene using the 2-parameter Kimura model with 1000 bootstrap replicates using MEGA6 software (Kimura, 1980; Tamura et al., 2011).

The most appropriate evolutionary model of nucleotide substitution was TPM3uf + G for the *28S* gene and TVM + I + G for the *ITS1* gene, as determined by the Akaike’s information criterion in the jModelTest program (Posada, 2008). The phylogenetic trees were constructed using maximum likelihood (ML) methods with RAxML (Guindon and Gascuel, 2003) and Bayesian inference (BI) with MrBayes (Ronquist and Huelsenbeck, 2003). Both analyses were performed on the CIPRES Science Gateway online platform (Miller et al., 2010).

Bayesian analyses employed the following settings for the ITS1 dataset: Iset nst = 6, rates = invgamma, ngammacat = 4, nucmodel = 4by4, code = universal, prset statefreqpr = dirichlet (1,1,1,1), shape = estimate, inferrates = yes, and basefreq = empirical. For the 28S analyses, Bayesian methods were applied with the following settings for the dataset: Iset nst = 6, rates = gamma, ngammacat = 4, nucmodel = 4by4, code = universal, prset statefreqpr = dirichlet (1,1,1,1), shape = estimate, inferrates = yes, and basefreq = empirical.

For the Markov Monte Carlo chain, chains with 10 000 00 generations were executed, and 1 tree was saved every 1500 generations. The first 25% of the generations were discarded as burn-in, and the consensus tree (majority rule) was estimated using the other topologies and we added commands sumt relburnin = yes, and sump relburnin = yes. Sampling adequacy was evaluated using Tracer v1.7.2. (Rambaut et al., 2018) to compute the effective sample sizes (ESSs) for the parameters. Values exceeding 200 effective independent samples were deemed robust. The ITS Bayesian sampling, after 25% burn-in, resulted in a mean Lnl = −2923.6887 score (standard deviation = 4.9649; median = −2923.35); Programmed ribosomal frameshifting (PRF)  + = 1.0. The ESSs were robust for all parameters. The 28S Bayesian sampling, after 25% burn-in, resulted in a mean Lnl = − 2635.0798 score (standard deviation = 5.1926; median = −2634.755); PRF + = 1.0. The ESSs were robust for all parameters.

Only nodes with posterior probabilities greater than 90% were considered credible. Maximum likelihood was implemented using bootstrap support values of 1000 repetitions, and only nodes with bootstrap values greater than 70% were considered well-supported. The trees were visualized and edited using FigTree v1.3.1 software (Rambaut, 2009).

### Map of occurrence of A. membranosa

We searched for bibliographic references and records in the Helminthological Collection database of the Instituto Oswaldo Cruz, Brazil (http://chioc.fiocruz.br/catalogue), to compile records of *A. membranosa* and prepare a distribution map of the species. The map was generated using a spreadsheet and QGIS 3.28 software (Quantum, 2024). This compilation included published records in South America, available data and information from the present study.

## Results

Taxonomic Summary

Family Cosmocercidae

Genus *Aplectana* Railliet and Henry, 1916

*Aplectana membranosa* (Schneider, 1866) Miranda, 1924

Type host: *Leptodactylus latrans* (Steffen, 1815) (=*Leptodactylus ocellatus*)

Additional hosts: *Leptodactylus pentadactylus* (Laurenti, 1768); *Leptodactylus labyrinthicus* (Spix, 1824); *Leptodactylus elenae* Heyer, 1978; *Scinax ruber* (Laurenti, 1768)

Neotype locality: Manguinhos, Rio de Janeiro, Brazil

Site of infection: Intestine

Neotypes: CHIOC 1593 and CHIOC 1594.

Voucher material: MPEG 293; MPEG 294; MPEG 295; MPEG 296; MPEG 297; MPEG 298; MPEG 299; MPEG 300; MPEG 301; MPEG 302; MPEG 303; MPEG 304; MPEG 305; MPEG 306; MPEG 307; MPEG 308; MPEG 309; MPEG 310; MPEG 311; MPEG 312; MPEG 313; MPEG 314.

Additional localities: Belém, Pará; Barro, Ceará; Barras, Piauí; Brasilândia, Mato grosso do Sul; FLONA Caxiuanã, Pará; Farias de Brito, Ceará; Macapá, Amapá.

GenBank Accession number: PQ569941; PQ569939; PQ569940; PQ592008; PQ580741.

**Figure 1.**
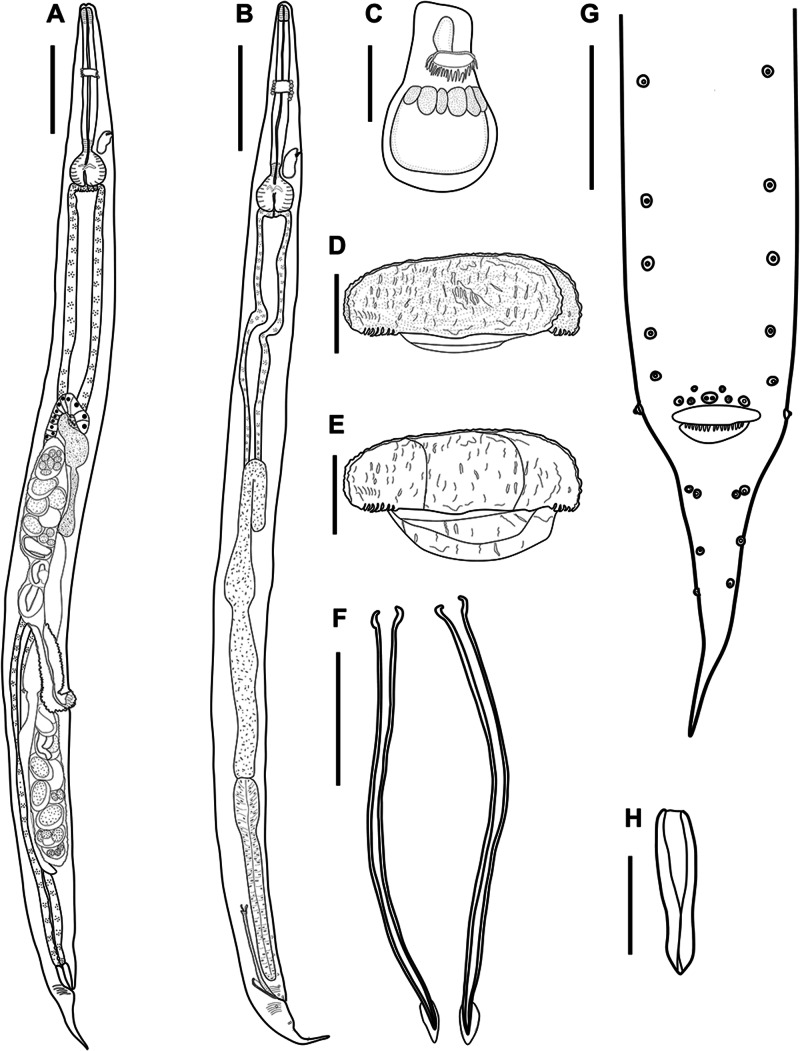
Line drawing of *A. membranosa* from Brazil. (A) Female, general overview, lateral view; (B) Male, general overview, lateral view; (C) Male, excretory pore, ventrolateral view; (D) Female, slight prominence of the lower vulva lip; (E) Female, greater prominence of the lower vulva lip; (F) Male, spicules, ventral view; (G) Male, caudal papilla pattern, lateral view; (H) Male, gubernaculum, ventral view. Scale bars: A, B – 200 µm; C, D, E, H – 30 µm; F, G – 50 µm.

**Figure 2.**
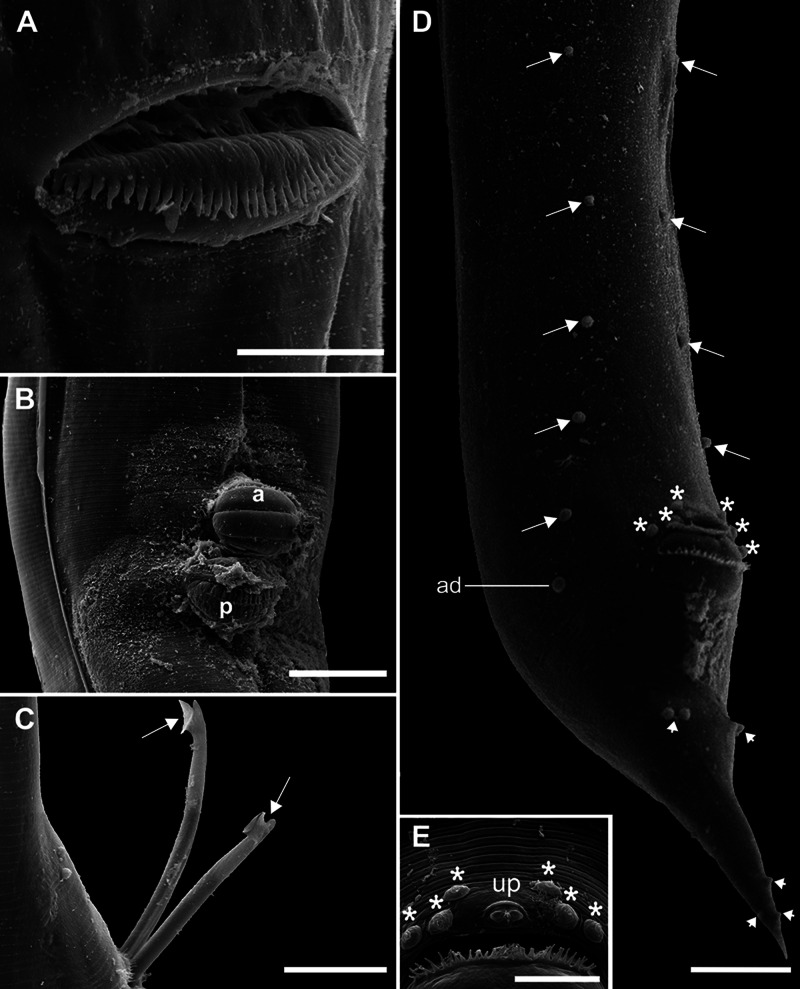
Scanning electron microscopy of *A. membranosa* from Brazil. (A) Male, excretory pore, showing the fringes; (B) Females, vulva view; (C) Male, spicules with bifid membrane; (D) Male, showing the pattern of pre-cloacal papillae (arrow), adcloacal papilla (ad), post-cloacal papillae (arrowhead); (E) Male, unpaired papilla (up), papillae on the upper lip of the cloaca (*). Scale bars: A, E – 10 µm, B – 25 µm, C – 50 µm, D – 30 µm.

Description (Figures 1–2)

Small nematodes, with transversal striations (Figure 2). Mouth triangular with 3 lips, each of them with cuticular flap on anterior edge. Dorsal lip with 2 papillae; ventrolateral lip with 1 ventral papilla and 1 lateral amphid. Oesophagus divided into anterior pharyngeal portion, elongate corpus, short and narrow isthmus, and large valved bulb. Evident excretory pore with fringe, near isthmus (Figures 1C; 2A). Lateral alae present in both sexes beginning at level of pharyngeal region and ending at level of anus in females and before the cloaca in males. *Females*: Vulva postequatorial, with 2 mamelon-like cuticular protuberance, located on each vulvar lip, the mamelon-like of the lower lip is smaller than that of the upper lip (Figures 1A, D, E; 2B). Well-developed ovojector (Figure 1A). Both ovaries directed anteriorly and flexed posteriorly to vulva; Uterus with numerous thin-shelled eggs (Figure 1A). *Males*: Caudal papillae of number and arrangement, divided into 3 groups: precloacal, adcloacal and postcloacal (Figure 2D), with the large unpaired papilla anterior to the cloaca. The caudal papillae consisted of 5 pairs of precloacal papillae, 1 pair of ad-cloacal papillae and 3 pairs of superior papillae at the fringed cloacal lip, with an odd papilla situated between them (Figure 2E), 4 pairs of postcloacal papillae (2 pairs ventrolaterally and adjacent and 2 pairs laterally, the latter located between 2 papillae) (Figures 1G; Figures. 2D, E). Gubernaculum long with ventral concavity (Figure 1H). Spicules comparatively long with a membrane on the distal end with a cup-like shape, that may have a bifurcated appearance (Figure 2C). Posterior edge of cloaca in males with comb-like cuticular fringe (Figure 2D, E). The measurements of the characteristics are shown in Table 3.

**Table 3.** Metrical characters of males and females of *A. membranosa* parasites of amphibians from the present study and reported by other authors from Brazil [mean ± sd (range)]

Locality (author)	Belém, Pará (Present study)						Caxiuanã, Pará (Present study)		Barras, Piauí (Present study)	
Host	*Rhinella marina*		*Leptodactylus fuscus*		*Leptodactylus paraensis*		*R. marina*		*Leptodactylus fuscus*	
	Females (*n* = 10)	Males (*n* = 10)	Females (*n* = 10)	Males (*n* = 10)	Females (*n* = 10)	Males (*n* = 10)	Females (*n* = 10)	Males (*n* = 10)	Females (*n* = 10)	Males (*n* = 10)
Total length (mm)	3.2 ± 0.42(2.5–3.8)	2.09 ± 0.26(1.9–2.6)	2.04 ± 0.25(1.7–2.4)	1.62 ± 0.21(1.2–1.9)	2.87 ± 0.19(2.4–3)	2.46 ± 0.22(2.2–2.9)	3.3 ± 0.26(3–3.9)	2.47 ± 0.22(2–2.7)	2.8 ± 0.25(2.4–3.2)	2.4 ± 0.9(2.6–3.1)
Width at oesophagus-intestine	160 ± 43(118–257)	105 ± 18(83–132)	132 ± 15(112–149)	102 ± 18(80–133)	165 ± 18(129–187)	110 ± 9(93–120)	195 ± 18(176–224)	127 ± 12(107–144)	123 ± 8(107–133)	97 ± 3(91–104)
Oesophagus length	511 ± 27(484–573)	460 ± 33.4 (376–494)	449 ± 22(422–484)	402 ± 23(366–429)	609 ± 25(561–642)	507 ± 16(469–534)	623 ± 21(597–663)	525 ± 27(460–560)	512 ± 37(453–579)	480 ± 17(458–515)
Corpus length	347 ± 21(320–396)	330 ± 11(313–344)	289 ± 19(264–320)	266 ± 17(237–285)	424 ± 18(392–469)	361 ± 16(331–387)	443 ± 20(416–469)	364 ± 33 (301–397)	351 ± 18(320–396)	328 ± 19(304–365)
Corpus width	45 ± 4(40–53)	36 ± 4(31–44)	35 ± 4(29–40)	29 ± 2(27–34)	46 ± 4(43–53)	32 ± 3(29–37)	46 ± 3(40–51)	37 ± 5(29–48)	36 ± 5(29–45)	32 ± 2(27–37)
Pharynx length	36 ± 4(32–44)	32 ± 3(26–35)	39 ± 3(35–45)	30 ± 4(24–35)	44 ± 2(40–48)	34 ± 4(27–40)	45 ± 5(39–56)	45 ± 7(32–53)	40 ± 3(35–45)	37 ± 2(32–40)
Pharynx width	29 ± 5(24–43)	21 ± 1(19–23)	27 ± 1(25–29)	20 ± 2(16–27)	25 ± 2(19–29)	21 ± 2(17–24)	30 ± 2(24–35)	23 ± 3(19–28)	25 ± 1(21–28)	21 ± 2(19–27)
Isthmus length	34 ± 3(30–42)	32 ± 4(25–38)	34 ± 5(25–49)	35 ± 5(29–40)	40 ± 8(29–53)	35 ± 5(27–40)	39 ± 4(30–45)	32 ± 2(28–35)	33 ± 4(27–40)	34 ± 4(27–40)
Isthmus width	29 ± 5(22–40)	23 ± 2(19–27)	25 ± 3(21–29)	24 ± 1(19–24)	30 ± 2(27–48)	24 ± 3(21–32)	35 ± 5(27–47)	24 ± 4(16–29)	27 ± 1(27–29)	24 ± 2(21–29)
Bulb length	90 ± 6(83–103)	75 ± 5(66–81)	84 ± 6(77–96)	72 ± 3(65–77)	98 ± 6(83–107)	77 ± 3(69–83)	96 ± 6(85–107)	79 ± 2(75–83)	92 ± 6(72–107)	81 ± 3(73–88)
Bulb width	94 ± 7(83–112)	75 ± 7(69–87)	87 ± 6(75–97)	67 ± 5(59–80)	98 ± 8(85–109)	71 ± 3(67–75)	106 ± 5(96–115)	78 ± 4(69–85)	91 ± 3(80–96)	71 ± 4(64–77)
Nerve ring^a^	225 ± 14(218–254)	237 ± 38(211–338)	174 ± 5(168–200)	165 ± 12(141–187)	245 ± 17(221–280)	220 ± 11(203–235)	264 ± 9(232–280)	244 ± 12(216–256)	228 ± 24(195–267)	205 ± 14(187–240)
Excretory pore^a^	365 ± 10(354–407)	324 ± 42(215–349)	310 ± 15(293–347)	285 ± 24(245–320)	468 ± 36(427–553)	384 ± 21(347–421)	504 ± 17(472–531)	433 ± 24(360–456)	399 ± 22(360–443)	360 ± 17(323–383)
Tail length	177 ± 31(151–249)	175 ± 23(132–205)	185 ± 17(152–213)	144 ± 13(129–162)	134 ± 6(127–145)	126 ± 20(116–178)	172 ± 17(108–232)	148 ± 13(131–171)	160 ± 9(145–175)	130 ± 7(119–143)
Vulva (mm)^b^	1.8 ± 0.23 (1.6–2.5)	–	1.28 ± 0.20(0.9–1.6)	–	2.02 ± 0.19(1.6–2.3)	–	2.2 ± 0.19(1.9–2.7)	–	2 ± 0.15(1.5–2.2)	–
Egg length	89 ± 5(75–88)	–	77 ± 9(61–89)	–	67 ± 3(61–70)	–	50 ± 2(46–53)	–	54 ± 3(48–58)	–
Egg width	55 ± 3(50–61)	–	52 ± 6(42–62)	–	39 ± 3(35–40)	–	36 ± 2(32–40)	–	37 ± 2(34–41)	–
Gubernaculum length	–	70 ± 8(49–79)	–	53 ± 6(48–61)	–	48 ± 5(38–53)	–	57 ± 2(43–61)	–	48 ± 5(42–60)
Spicule length	–	203 ± 13(169–215)	–	172 ± 10(48–56)	–	177 ± 10(162–197)	–	202 ± 17(175–238)	–	156 ± 5(143–162)

Locality (author)	Macapá, Amapá (Present study)						Barro, Ceará (Present study)			
Host	*Rhinella major*		*R. marina*		*Leptodactylus fuscus*		*Leptodactylus fuscus*			
	Females (*n* = 10)	Males (*n* = 10)	Females (*n* = 10)	Males (*n* = 10)	Females (*n* = 10)	Males (*n* = 10)	Females (*n* = 10)		Machos (*n* = 10)	
Total length (mm)	3.1 ± 0.37(2.5–3.8)	2.5 ± 0.18(2.1–2.7)	3.4 ± 0.26(2.8–3.7)	2.88 ± 0.11(2.7 – 3.1)	2.5 ± 0.34 (2.4–3.2)	2.1 ± 0.10(1.9–2.3)	2.65 ± 0.38(2.3–3.4)		2 ± 0.11(1.8–2.2)	
Width at oesophagus-intestine	155 ± 23(109–195)	99 ± 5(83–112)	172 ± 13(141–187)	116 ± 12(99–141)	139 ± 18(109–171)	106 ± 9(83–125)	121 ± 16(92–144)		90 ± 10(73–102)	
Oesophagus length	586 ± 38(523–635)	494 ± 27(454–539)	622 ± 12(603–653)	523 ± 13(499–538)	502 ± 51(461–600)	432 ± 11(395–448)	494 ± 57(454–642)		425.67 ± 10(408–441)	
Corpus length	409 ± 27(360–440)	346 ± 23(304–381)	435 ± 13(408–464)	367 ± 10(344–376)	335 ± 39(304–405)	290 ± 7(264–304)	331 ± 18(304–365)		293.53 ± 10(279–309)	
Corpus width	42 ± 4(37–54)	29 ± 3(19–35)	42 ± 2(37–45)	31 ± 3(24–37)	39 ± 2(29–43)	29 ± 2(24–35)	38 ± 4(29–48)		30 ± 2(26.34)	
Pharynx length	46 ± 6(37–54)	37 ± 1(35–40)	44 ± 6(29–51)	37 ± 4(27–43)	40 ± 2(32–43)	37 ± 2(32–40)	34 ± 3(27–41)		30 ± 1(24–32)	
Pharynx width	26 ± 3(21–29)	20 ± 1(16–21)	27 ± 1(21–29)	22 ± 2(18–27)	28 ± 2(21–29)	19 ± 2(16–24)	25 ± 2(16–27)		19 ± 2(15–22)	
Isthmus length	34 ± 3(29–40)	33 ± 4(29–40)	39 ± 7(29–53)	36 ± 3(27–40)	34 ± 5(29–45)	32 ± 3(21–35)	35 ± 2(31–40)		33 ± 3(29–37)	
Isthmus width	26 ± 1(19–29)	21 ± 3(16–27)	27 ± 1(24–29)	23 ± 2(19–27)	24 ± 3(21–29)	19 ± 1(16–19)	26 ± 1(21–28)		20 ± 2(18–24)	
Bulb length	98 ± 6(85–107)	77 ± 4(72–83)	109 ± 7(93–123)	86 ± 4(77–91)	93 ± 8(85–112)	75 ± 6(64–88)	84 ± 7(77–103)		70 ± 4(63–76)	
Bulb width	106 ± 9(91–120)	72 ± 4(67–80)	108 ± 8(96–120)	79 ± 5(67–85)	92 ± 12(80–121)	72 ± 7(53–80)	91 ± 8(77–102)		69 ± 5(55–77)	
Nerve ring^a^	258 ± 34(208–312)	222 ± 15(200–253)	249 ± 5(235–259)	219 ± 11(200–232)	242 ± 24(197–288)	207 ± 15(184–240)	225 ± 19(200–269)		205 ± 5(189–213)	
Excretory pore^a^	453 ± 36(395–523)	389 ± 13(360–400)	475 ± 14(448–496)	412 ± 9(392–424)	379 ± 48(339–469)	326 ± 6(320–339)	363 ± 44(326–478)		316 ± 12(285–331)	
Tail length	168 ± 14(134–194)	164 ± 11(126–188)	183 ± 17(160–221)	183 ± 14(167–210)	149 ± 11(131–164)	136 ± 10(116–156)	173 ± 14(142–207)		161 ± 8(134–174)	
Vulva (mm)^b^	2.2 ± 0.29(1.7–2.7)	–	2.39 ± 0.17(2.2–2.7)	–	1.7 ± 0.26(1.5–2.2)	–	1.7 ± 0.22(1.6–2.3)		–	
Egg length	72 ± 5(61–79)	–	71 ± 2(66–74)	–	81 ± 5(55–88)	–	77 ± 6(68–86)		–	
Egg width	44 ± 3(38–51)	–	44 ± 2(40–47)	–	46 ± 2(31–49)	–	50 ± 3(45–58)		–	
Gubernaculum length	–	68 ± 5(55–79)	–	73 ± 5(60–81)	–	56 ± 3(48–61)	–		66 ± 2(55–66)	
Spicule length	–	217 ± 12(196–236)	–	219 ± 7(208–236)	–	161 ± 9(145–182)	–		189 ± 5(177–197)	

Locality (author)	Farias Brito-CE (Present study)									
Host	*Rhinella granulosa*		*Leptodactylus troglodytes*		*Leptodactylus syphax*		*Leptodactylus vastus*			
	Females (*n* = 10)	Males (*n* = 10)	Females (*n* = 10)	Males (*n* = 10)	Females (*n* = 10)	Males (*n* = 10)	Females (*n* = 10)		Males (*n* = 10)	
Total length (mm)	2.8 ± 0.25(2.4–3.2)	2.5 ± 0.36(2–3)	2.6 ± 0.15(2.5–2.8)	2.3 ± 0.20(2–2.6)	3.1 ± 0.34(2.8–3.7)	2.9 ± 0.30(2.4–3.3)	2.7 ± 0.11(2.6–3)		2.5 ± 0.16(2.3–2.7)	
Width at oesophagus-intestine	123 ± 8(107–133)	103 ± 10(85–120)	116 ± 16(93–144)	96 ± 9(80–109)	122 ± 7(112–134)	110 ± 7(97–123)	120 ± 11(99–141)		96 ± 13(83–129)	
Oesophagus length	524 ± 32(579)	455 ± 29(402–501)	533 ± 14(499–552)	472 ± 12(424–483)	623 ± 35(578–671)	564 ± 34(512–606)	601 ± 10(578–619)		530 ± 17(506–559)	
Corpus length	351 ± 18(320–396)	311 ± 21(275–352)	368 ± 12(336–381)	325 ± 9(283–333)	449 ± 30(397–489)	408 ± 32(355–452)	432 ± 7(416–446)		382 ± 14(360–410)	
Corpus width	33 ± 3(27–37)	27 ± 2(24–31)	37 ± 4(29–48)	31 ± 3(26–37)	40 ± 3(33–45)	35 ± 4(27–43)	43 ± 3(36–48)		36 ± 0.52(36–37)	
Pharynx length	40 ± 3(35–45)	35 ± 4(29–40)	41 ± 2(37–45)	35 ± 2(31–37)	42 ± 3(34–45)	34 ± 2(29–37)	36 ± 3(30–42)		31 ± 3(27–37)	
Pharynx width	25 ± 1(21–28)	22 ± 3(24–31)	23 ± 2(19–27)	20 ± 1(16–24)	27 ± 1(24–30)	22 ± 1(20–24)	25 ± 1(22–29)		20 ± 2(16–23)	
Isthmus length	33 ± 4(27–40)	33 ± 3(29–40)	36 ± 3(29–40)	31 ± 3(27–35)	38 ± 2(29–41)	33 ± 3(28–37)	35 ± 3(29–41)		35 ±(31–42)	
Isthmus width	27 ± 1(24–29)	21 ± 2(17–24)	25 ± 2(21–29)	21 ± 2(17–24)	29 ± 0.93(27–30)	25 ± 2(21–27)	27 ± 1(24–30)		22 ± 2(20–27)	
Bulb length	92 ± 6(72–107)	77 ± 3(65–80)	90 ± 3(80–96)	83 ± 3(72–85)	98 ± 4(91–106)	90 ± 4(83–96)	99 ± 2(92–103)		82 ± 5(81–91)	
Bulb width	91 ± 3(80–96)	68 ± 4(64–77)	89 ± 8(72–107)	73 ± 6(64–85)	96 ± 4(88–103)	86 ± 5(77–95)	95 ± 4(85–100)		79 ± 7(71–95)	
Nerve ring^a^	228 ± 24(195–267)	206 ± 20(179–240)	222 ± 9(213–240)	212 ± 17(176–240)	259 ± 24(227–303)	240 ± 19(208–271)	249 ± 9(228–260)		238 ± 5(230–246)	
Excretory pore^a^	399 ± 22(360–443)	364 ± 31(315–427)	401 ± 9(381–413)	363 ± 12(309–381)	466 ± 34(400–504)	435 ± 33(379–473)	455 ± 11(423–470)		413 ± 14(394–433)	
Tail length	160 ± 9(145–175)	168 ± 13(148–183)	136 ± 11(110–158)	129 ± 8(117–144)	179 ± 13(165–202)	182 ± 24(155–234)	156 ± 11(136–180)		151 ± 9(137–166)	
Vulva (mm)^b^	1.98 ± 0.17(1.7–2.2)	–	1.8 ± 0.11(1.7–2.1)	–	2 ± 0.36(1.05–2.4)	–	2 ± 0.06(1.9–2.1)		–	
Egg length	60 ± 2(57–65)	–	54 ± 2(50–58)	–	57 ± 8(42–65)	–	58 ± 3(50–62)		–	
Egg width	37 ± 1(33–40)	–	35 ± 1(32–37)	–	36 ± 2(30–38)	–	40 ± 1(37–43)		–	
Gubernaculum length	–	58 ± 6(39–69)	–	44 ± 5(39–56)	–	63 ± 6(52–71)	–		53 ± 3(49–59)	
Spicule length	–	194 ± 21(156–234)	–	151 ± 7(136–162)	–	211 ± 25(169–243)	–		165 ±(156–171)	

Locality (author)	Manguinhos, Rio de Janeiro (Miranda, 1924)		Brasil (Travassos, 1931)		Salvador, Bahia (Fahel, 1952)				Manaus, Amazonas (Gonçalves et al., 2002)	
Host	*Leptodactylus latrans*		*Leptodactylus latrans; Rhinella marina*		*Leptodactylus latrans*		*Leptodactylus pentadactylus*		*Rhinella marina; Rhinella granulosa*	
	Females (nd)	Males (nd)	Females (nd)	Males (nd)	Females	Males (*n* = 6)	Females (*n* = 3)	Males (*n* = 6)	Females (*n* = 9)	Males (*n* = 12)
Total length	3–3.5	2–2.5	2.4–3.5	2–2.6	nd	2.63 ± 0.29 (2.4–3.2)	3.1 ± 0.64 (2.8–3.9)	2.7 ± 0.65 (2–3.6)	2.17–4.77	1.93–3.17
Width at oesophagus-intestine	400	174	220	170–210	nd	151.7 ± 16 (140–180)	193.3 ± 30.5 (160–220)	128.3 ± 63.3 (70–240)	150	120–260
Oesophagus length	nd	nd	380–400	360–440	nd	530 ± 62 (430–600)	643.3 ± 68 (590–720)	525 ± 52 (480–600)	370 – 620	440–600
Corpus length	333.7	319.5	nd	nd	nd	nd	nd	nd	nd	nd
Corpus width	42.6	42.6	nd	nd	nd	nd	nd	nd	nd	nd
Pharynx length	49.7	44	44–56	nd	nd	33 ± 0 (33–33)	41	nd	30–60	30–50
Pharynx width	nd	nd	nd	nd	nd	nd	nd	nd	nd	nd
Isthmus length	85.2	78	nd	nd	nd	nd	nd	nd	nd	nd
Isthmus width	14.2	14	nd	nd	nd	nd	nd	nd	nd	nd
Bulb length	nd	nd	120–280	nd	nd	107.3 ± 7.6 (100–116)	132 ± 8 (124–140)	110 ± 45.4 (49–184)	80–120	–
Bulb width	142	85.2	80–140	120–280	nd	81 ± 9.1 (74–93)	107	85	130	70–100
Nerve ring^a^	227.7	213	nd	210–240	nd	212 ± 16.4 (190–230)	240	nd	100–250	100–250
Excretory pore^a^	390.5	263	nd	nd	nd	407.5 ± 12.6 (390–420)	460	nd	nd	nd
Tail length	213	177.5	200–210	nd	nd	120 ± 31.6 (80–160)	171 ± 12.7 (157–182)	160 ± 28.2 (140–180)	nd	150–250
Vulva (mm)^b^	2.52	–	2	–	nd	–	1.95 ± 0.07 (1.9–2)	–	1.47–3.64	–
Egg length	nd	–	96	–	nd	–	75.6 ± 2.89 (74–79)	–	60–100	–
Egg width	nd	–	56	–	nd	–	43.6 ± 4.6 (41–49)	–	30–50	–
Gubernaculum length	–	71	–	71–80	nd	53.2 ± 9.4 (41–66)	–	65 ± 15.7 (49–95)	–	50–90
Spicule length	–	227–243	–	220–230	nd	184 ± 15.7 (166–212)	–	194.8 ± 27.9 (152–232)	–	200–280

a From the anterior end.

b From the posterior end.

### Morphological variation of different hosts and localities

We analysed 130 males and 130 females of *A. membranosa* from different hosts and locations in Brazil. We also analysed Miranda’s (1924) specimens deposited in the Helminthological Collection of the Oswaldo Cruz Institute.

We observed that females had 2 protuberances on the vulvar lips (Figure 2B), 1 more on the upper lip, and 1 on the lower lip of the vulva (Figure 1D, E). We did not observe variation in the number of protuberances according to host or location. However, in some specimens, the lower lip protuberance was more discreet, especially when it was observed in the dorsoventral view, and may go unnoticed.

In males, the morphology of spicules and gubernaculum, the number and pattern of the caudal papillae did not vary in according to host or location. All presented 2 subequal long spicules with a membrane, which, when observed in lateral view, shows a bifurcated aspect at the distal end (Figure 2C). When observed in dorsoventral view, this membrane has a cup-like shape. The gubernaculum was concave and well sclerotized in all specimens analysed (Figure 1H).

### Variation of metric characters

Table 4 shows the PCA and the percentage of variance of the morphometric variables of the *A. membranosa* females (*n* = 130). The first axis (PCA1) explained 44.18% of the observed variation, highlighting the influence of corpus length, bulb length and width, distance from nerve ring, from excretory pore to anterior end, and from the vulva to posterior end. The second axis (PCA2) explained 16.62% of the variation, emphasising the influence of tail length and egg length and width. The combined value of both axes was 60.80%.

**Table 4.** Results of principal component analysis of morphometric characters of females of *A. Membranosa* (*n* = 130): Coefficients for standardized measurements and percentage of explained variation

	PCA1	PCA2
Total length	0.3172454	0.02499802
Width at oesophagus-intestine	0.3694818	0.34504965
Oesophagus length	0.8201145	−0.17762911
Pharynx length	0.4300642	−0.06928452
Pharynx width	0.3812086	0.59394335
Corpus length	1.0697507	−0.30586014
Corpus width	0.7852243	0.58079509
Isthmus length	0.4755671	0.02092157
Isthmus width	0.8718018	0.33219302
Bulb length	1.1386358	0.10356771
Bulb width	1.2770304	0.82379923
Nerve ring^a^	1.6514708	−0.31041180
Excretory pore^a^	2.3522026	−0.66759913
Tail length	0.3540340	1.83153047
Vulva^a^	3.7901199	−0.22060997
Egg length	−1.7493028	3.29191192
Egg width	−2.6884740	5.63286788
Eigenvalue	7.51074881	2.82573891
Percentage of total variance explained	44.1808754	16.6219936
Cumulative percentage	44.18088	60.80287

a From the anterior end.

Table 5 shows the PCA and the percentage of variance of the morphometric variables of the males of *A. membranosa* (*n* = 130). The first axis (PCA1) explained 40.61% of the observed variation, showing the influence of total body length, oesophageal length, corpus length and distance from the excretory pore to the anterior end, on the morphometric variation of *A. membranosa* males. In comparison, the second axis (PCA2) explained 13.37% of the morphometric variation, highlighting the influence of tail length in relation to the posterior end, gubernaculum size and spicule size. The combined value of both axes was 53.99%.

**Table 5.** Results of principal component analysis of morphometric characters of males of *A. Membranosa* (*n* = 130): coefficients for standardized measurements and percentage of explained variation

	PCA1	PCA2
Total length	0.332	0.011
Width at oesophagus-intestine	0.235	−0.024
Oesophagus length	0.363	−0.110
Pharynx length	0.145	−0.178
Pharynx width	0.151	−0.087
Corpus length	0.351	−0.089
Corpus width	0.184	−0.111
Isthmus length	0.068	0.063
Isthmus width	0.177	−0.198
Bulb length	0.291	−0.117
Bulb width	0.299	−0.065
Nerve ring^a^	0.286	−0.040
Excretory pore^a^	0.332	−0.147
Tail length	0.185	0.515
Gubernaculum length	0.139	0.578
Spicule length	0.206	0.491
Eigenvalue	6.498	2.140
Percentage of total variance explained	40.61	13.37
Cumulative percentage	40.61	53.99

a From anterior end.

Females of *A. membranosa* from different host species and different localities exhibited significant differences in all morphometric comparisons (Females: host species: MANOVA Pillai = 2.216; F = 5.06; *P* < 0.00; locality: MANOVA Pillai=1.68; *F* = 6.74; P<0.00) (Table 6). Males of *A. membranosa* also showed significant differences in all morphometric comparisons from different host species and different localities (Males: host species: MANOVA Pillai = 2.23; *F* = 8.15; *P* < 0.00; locality: MANOVA Pillai = 1.47; *F* = 7.32; *P* < 0.00) (Table 7).

**Table 6.** Summary of 1-way analysis of female morphological characters of *A. membranosa*, anuran hosts and localities

	Anuran host		Locality	
	*F*	*P*	*F*	*P*
Corpus length	27.06	<0.000	8.52	0.000
Bulb length	5.63	0.000	7.41	0.000
Bulb width	7.48	0.000	11.06	0.000
Nerve ring^a^	6.73	0.000	7.52	0.000
Excretory pore^a^	17.34	<0.000	14.33	0.000
Tail length	10.29	0.000	4.88	0.000
Vulva^a^	12.51	0.000	6.34	0.000
Egg length	13.11	0.000	59.91	<0.000
Egg width	13.11	0.000	28.74	<0.000

a From the anterior end.

**Table 7.** Summary of 1-way analysis of variance of male morphological characters of *A. membranosa*, anuran hosts and localities

	Anuran host		Locality	
	*F*	*P*	*F*	*P*
Total length	21.80	<0.000	10.29	0.000
Oesophagus length	42.52	<0.000	8.20	0.000
Corpus length	40.41	<0.000	6.42	0.000
Excretory pore^a^	18.92	<0.000	15.29	0.000
Tail length	16.68	0.000	3.24	0.000
Gubernaculum length	20.40	<0.000	7.72	0.000
Spicule length	38.56	<0.000	6.34	0.000

a From the anterior end.

The post hoc Tukey test revealed differences in at least 1 morphological trait in females or males of *A. membranosa* between all possible pairs of host amphibian species besides the pairs *R. major/L. fuscus, R. major*/*L. paraensis, R. major/L. sypax, L. vastus/L. troglodytes, R. granulosa/L. troglodytes, R. major/L. troglodytes, R. major/L. vastus* and *R. marina/R. major* in the case of female nematodes (Table 8) and besides the pairs *R. granulosa*/*L. paraensis* and *R. marina/R. major* in the case of male (Table 9).

**Table 8.** Host pairs comparison of selected morphological characters of females of *A. membranosa* showing the *p*-values

Hospedeiro	Corpus length	Bulb length	Bulb width	Nerve ring^a^	Excretory pore^a^	Tail length	Vulva^a^	Egg length	Egg width
*L. paraenses*/*L. fuscus*	**0.0000000**	**0.0168350**	0.6436053	**0.0076530**	**0.0000000**	**0.0010062**	**0.0000154**	0.1622497	**0.0002447**
*L. syphax*/*L. fuscus*	**0.0000000**	**0.0078180**	0.8806423	**0.0000667**	**0.0000000**	0.7172517	**0.0000227**	**0.0000001**	**0.0000000**
*L. troglodytes*/*L. fuscus*	**0.0007120**	0.9888664	0.9777551	0.9369182	**0.0068625**	**0.0002939**	**0.0166890**	**0.0000000**	**0.0000000**
*L. vastus*/*L. fuscus*	**0.0000000**	**0.0162369**	0.9995065	**0.0005486**	**0.0000000**	**0.0010331**	**0.0000012**	**0.0000000**	**0.0000019**
*R. granulosa*/*L. fuscus*	0.0920962	0.9271336	0.9999996	0.7715607	**0.0184857**	0.9940662	**0.0002368**	**0.0003136**	**0.0000015**
*R. major/L. fuscus*	0.5936804	0.9999995	1.0000000	0.9794438	0.7002040	0.9969211	0.4661962	0.9963202	0.8115479
*R. marina*/*L. fuscus*	**0.0000000**	**0.0000039**	**0.0000280**	**0.0000036**	**0.0000000**	0.7537728	**0.0000000**	**0.0027504**	0.1806626
*L. syphax*/*L. paraensis*	0.9955414	0.9999999	0.9999942	0.9842806	0.9999989	**0.0000799**	1.0000000	0.0791714	0.7410320
*L. troglodytes*/*L. paraensis*	**0.0028478**	0.4803139	0.2999624	0.5219914	0.1333545	0.9999994	0.8618927	**0.0127808**	0.5270702
*L. vastus*/*L. paraensis*	0.7538683	0.9988653	0.4067911	0.9999999	0.9946678	0.9945330	0.9993123	**0.0284155**	1.0000000
*R. granulosa*/*L. paraensis*	**0.0000213**	0.6751316	0.7360131	0.7307136	0.0719503	0.1188449	0.9998430	0.8123852	0.9897209
*R. major*/*L. paraensis*	0.8981819	0.9867979	0.9996630	0.9999984	0.9969808	0.9980665	0.9999999	1.0000000	1.0000000
*R. marina*/*L. paraensis*	0.7128250	0.9999330	0.6666716	0.9999997	0.9998814	**0.0000069**	0.9730723	1.0000000	0.1196794
*L. troglodytes*/*L. syphax*	**0.0001094**	0.3570129	0.5151335	0.0681000	0.0668171	**0.0000266**	0.8920233	0.9994418	0.9999965
*L. vastus*/*L. syphax*	0.1773879	0.9920591	0.6734449	0.9058587	0.9600836	**0.0001178**	0.9997529	1.0000000	0.5005401
*R. granulosa*/*L. syphax*	**0.0000005**	0.5445659	0.9058854	0.1472947	**0.0333712**	0.4541407	0.9999485	0.8936495	0.9976527
*R. major*/*L. syphax*	0.7144465	0.9780787	0.9999620	0.9984076	0.9922062	0.9051022	0.9999998	0.8864471	0.9958753
*R. marina*/*L. syphax*	0.1337670	0.9999997	0.3852030	0.9852904	0.9951741	0.9997711	0.9574180	**0.0194424**	**0.0000978**
*L. vastus*/*L. troglodytes*	0.0786876	0.7245305	0.9997835	0.4489093	0.3218821	0.9637954	0.9775151	0.9962078	0.2735822
*R. granulosa*/*L. troglodytes*	0.9511673	0.9999986	0.9990275	0.9999972	0.9999995	0.0617925	0.9889916	0.5263066	0.9757125
*R. major*/*L. troglodytes*	0.9999953	1.0000000	0.9999624	0.9996831	0.9999825	0.9949596	0.9959170	0.7514867	0.9884629
*R. marina*/*L. troglodytes*	**0.0436089**	0.0694134	**0.0001934**	0.1483776	0.1005229	**0.0000016**	0.0952638	**0.0014019**	**0.0000181**
*R. granulosa*/*L. vastus*	**0.0007807**	0.8946130	0.9999998	0.6925066	0.1832600	0.2924915	1.0000000	0.8403285	0.9576352
*R. major*/*L. vastus*	0.9969821	0.9979678	0.9999997	0.9999998	0.9999166	0.9999643	0.9999598	0.8874639	1.0000000
*R. marina*/*L. vastus*	1.0000000	0.8454154	**0.0000212**	0.9996845	0.9998775	**0.0000017**	0.3786879	**0.0012530**	**0.0218651**
*R. major*/*R. granulosa*	0.9948794	0.9999999	1.0000000	0.9999561	0.9998922	0.9999394	0.9999733	0.9959116	0.9998914
*R. marina*/*R. granulosa*	**0.0002354**	0.1634679	**0.0044634**	0.3221187	**0.0448757**	0.5032671	0.7174009	0.7091878	**0.0036571**
*R. marina*/*R. major*	0.9964330	0.9586679	0.9417394	0.9999856	0.9993833	0.9506408	0.9999999	0.9999998	0.9918496

Significant values are in bold.

a From the anterior end.

**Table 9.** Host pairs comparison of selected morphological characters of males of *A. membranosa* showing the *p*-values

Host	Total length	Oesophagus length	Corpus length	Excretory pore^a^	Tail length	Gubernaculum length	Spicule length
*L. paraenses*/*L. fuscus*	**0.0000013**	**0.0000000**	**0.0000000**	**0.0000010**	**0.0163273**	**0.0062998**	0.9871381
*L. syphax*/*L. fuscus*	**0.0000000**	**0.0000000**	**0.0000000**	**0.0000000**	**0.0000137**	0.3350117	0.0000000
*L. troglodytes*/*L. fuscus*	**0.0000382**	**0.0000200**	**0.0001140**	**0.0016224**	0.0766208	**0.0000012**	0.0000516
*L. vastus*/*L. fuscus*	**0.0000000**	**0.0000000**	**0.0000000**	**0.0000000**	0.9711058	**0.0392497**	**0.0210187**
*R. granulosa*/*L. fuscus*	**0.0000000**	**0.0230451**	**0.0344048**	**0.0014384**	**0.0314782**	0.9997543	0.0552914
*R. major/L. fuscus*	**0.0000002**	**0.0000000**	**0.0000000**	**0.0000029**	0.4585045	**0.0367655**	**0.0000000**
*R. marina*/*L. fuscus*	**0.0000000**	**0.0000000**	**0.0000000**	**0.0000000**	**0.0000861**	**0.0003429**	**0.0000000**
*L. syphax*/*L. paraensis*	**0.0186424**	**0.0007703**	**0.0045928**	0.0967647	**0.0000000**	**0.0000809**	**0.0001576**
*L. troglodytes*/*L. paraensis*	0.9985426	0.0968559	**0.0229305**	0.8454222	0.9998458	0.6881449	**0.0001182**
*L. vastus*/*L. paraensis*	1.0000000	1.0000000	0.9993492	0.9999999	0.2019823	0.9367966	**0.0229832**
*R. granulosa*/*L. paraensis*	0.9926917	**0.0007086**	**0.0002161**	0.8577279	**0.0000064**	0.1386150	0.6428705
*R. major*/*L. paraensis*	0.9999788	0.9682652	0.8090274	0.9999996	**0.0004142**	**0.0000026**	**0.0000743**
*R. marina*/*L. paraensis*	0.9969206	1.0000000	0.9988277	1.0000000	**0.0000000**	**0.0000000**	**0.0000243**
*L. troglodytes*/*L. syphax*	**0.0023104**	**0.0000000**	**0.0000000**	**0.0010549**	**0.0000000**	**0.0000000**	**0.0000000**
*L. vastus*/*L. syphax*	**0.0043693**	**0.0000566**	**0.0000543**	**0.0461779**	**0.0000017**	**0.0004467**	**0.0000000**
*R. granulosa*/*L. syphax*	0.1593306	**0.0000000**	**0.0000000**	**0.0011658**	0.6979432	0.3496935	0.0683440
*R. major*/*L. syphax*	**0.0488865**	**0.0000104**	**0.0000118**	0.0601688	0.1428220	0.9939734	0.9999997
*R. marina*/*L. syphax*	**0.0149251**	**0.0000107**	**0.0000135**	**0.0245210**	0.5827234	0.9776277	0.9985066
*L. vastus*/*L. troglodytes*	0.9925802	**0.0273503**	**0.0250236**	0.6222015	0.4868361	**0.0429205**	0.3616321
*R. granulosa*/*L. troglodytes*	0.8449620	0.7916215	0.8955744	1.0000000	**0.0000443**	**0.0006524**	**0.0000000**
*R. major*/*L. troglodytes*	0.9803477	0.6232713	0.5812116	0.9190023	**0.0022049**	**0.0000000**	**0.0000000**
*R. marina*/*L. troglodytes*	0.8280796	0.0190234	**0.0142229**	0.5685601	**0.0000001**	**0.0000000**	**0.0000000**
*R. granulosa*/*L. vastus*	0.9904996	**0.0000408**	**0.0001063**	0.6426687	**0.0050704**	0.5416638	**0.0000094**
*R. major*/*L. vastus*	0.9999907	0.9245414	0.9367737	0.9999531	0.1379105	**0.0000102**	**0.0000000**
*R. marina*/*L. vastus*	0.9945907	1.0000000	1.0000000	1.0000000	**0.0000098**	**0.0000000**	**0.0000000**
*R. major*/*R. granulosa*	0.9997514	0.0251051	**0.0400283**	0.9272540	0.9758915	0.0638826	**0.0417233**
*R. marina*/*R. granulosa*	0.9999920	**0.0000152**	**0.0000332**	0.5905938	0.9999992	**0.0079868**	0.0607590
*R. marina*/*R. major*	0.9999900	0.9263619	0.9212079	0.9999591	0.8510794	1.0000000	0.9900801

Significant values are in bold.

a From the anterior end.

The analyses of the morphometric variations of *A. membranosa* between pairs from different locations showed significant differences in at least 1 morphometric characteristic, except for the pairs Farias Brito – CE and Caxiuanã – PA, as well as Belém – PA and Barro – CE, which did not show significance in any characteristic (Tables 10 and 11). It was not possible to observe any case in which all characteristics showed statistical significance in all pairs.

**Table 10.** Locality pairs comparison of selected morphological characters of females of *A. membranosa* showing the *p*-values

Locality	Corpus length	Bulb length	Bulb width	Nerve ring^a^	Excretory pore^a^	Tail length	Vulva^a^	Egg length	Egg width
Barro – CE/Barras – PI	0.1141457	0.2289837	0.8192607	0.8755911	**0.0165153**	**0.0013108**	0.5559829	**0.0000000**	**0.0000120**
Belém – PA/Barras – PI	0.5664786	10.000.000	0.0531262	0.1910844	**0.0426489**	**0.0003948**	0.3029040	**0.0000000**	**0.0000102**
Caxiuanã–PA/Barras – PI	0.0892644	0.7605681	**0.0000222**	0.2723185	**0.0166506**	**0.0103216**	0.4813439	0.3666876	0.8149740
Farias Brito–CE/Barras – PI	0.9536627	0.8773791	0.1872022	0.9999898	0.9999999	**0.0102056**	0.9999542	0.7738040	0.9125804
Macapá – AP/Barras – PI	0.9989059	0.0632627	**0.0000033**	0.8924222	0.9999080	**0.0005145**	0.9532974	**0.0000000**	**0.0131918**
Belém–PA/Barro – CE	0.6718542	0.0671551	0.7503575	0.9279475	0.8940277	0.9916090	10.000.000	0.9884212	0.8675503
Caxiuanã – PA/Barro – CE	**0.0000102**	**0.0064210**	**0.0028315**	**0.0178940**	**0.0000000**	0.9907520	**0.0098017**	**0.0000000**	**0.0000000**
Farias Brito – CE/Barro – CE	**0.0009903**	**0.0018705**	0.9710347	0.6295424	**0.0006938**	0.5563066	0.1981573	**0.0000000**	**0.0000000**
Macapá – AP/Barro – CE	**0.0077035**	**0.0000025**	**0.0016474**	0.1534742	**0.0005839**	0.9861300	**0.0479672**	0.8485924	**0.0367022**
Caxiuanã – PA/Belém – PA	**0.0000394**	0.5992938	**0.0148166**	**0.0000342**	**0.0000000**	0.9999900	**0.0005515**	**0.0000000**	**0.0000000**
Farias Brito – CE/Belém – PA	**0.0034169**	0.6288086	0.9406059	**0.0037824**	**0.0001846**	0.6331819	**0.0099821**	**0.0000000**	**0.0000000**
Macapá – AP/Belém – PA	0.0524336	**0.0020845**	**0.0043876**	**0.0000637**	**0.0002171**	0.9999988	**0.0007792**	0.9726586	0.0921628
Farias Brito – CE/ Caxiuanã – PA	0.1298381	0.9914888	**0.0012156**	0.1145028	**0.0010519**	0.9443808	0.3189716	**0.0029386**	0.9934654
Macapá – AP/ Caxiuanã – PA	0.0514386	0.8545187	0.9808883	0.6294334	**0.0031912**	0.9999996	0.7770350	**0.0000000**	**0.0000471**
Macapá – AP/ Farias Brito – CE	0.9828075	0.1002292	**0.0000489**	0.7267944	0.9998000	0.6990542	0.9107117	**0.0000000**	**0.0000000**

a From the anterior end.t values are in bold.

**Table 11.** Locality pairs comparison of selected morphological characters of males of *A. membranosa* showing the *p*-values

Locality	Total length	Oesophagus length	Corpus length	Excretory pore^a^	Tail length	Gubernaculum length	Spicule length
Barro – CE/Barras – PI	0.1648901	0.0540034	0.3444731	0.1252978	**0.0350564**	**0.0032081**	**0.0112562**
Belém – PA/Barras – PI	0.0891237	0.7534365	0.9836958	0.3187663	0.2200318	0.0791315	**0.0047283**
Caxiuanã–PA/Barras – PI	0.9988383	0.3844079	0.3246192	**0.0102825**	0.4857688	0.2671432	**0.0003358**
Farias Brito–CE/Barras – PI	0.6823330	0.7299759	0.4307106	0.2494670	0.0113477	0.3969047	**0.0429702**
Macapá – AP/Barras – PI	0.9926720	10.000.000	0.9996951	0.9513713	**0.0097733**	**0.0000297**	**0.0000416**
Belém–PA/Barro – CE	0.9998053	0.2667089	0.4848260	0.9044828	0.6978881	0.3967894	0.9960833
Caxiuanã – PA/Barro – CE	0.0664287	**0.0000639**	0.4848261	**0.0000004**	0.8112904	0.5806752	0.9209649
Farias Brito – CE/Barro – CE	**0.0002151**	**0.0000256**	0.4848262	**0.0000052**	0.9982359	0.0535633	0.7527076
Macapá – AP/Barro – CE	**0.0080669**	**0.0075582**	0.4848263	**0.0016716**	0.9998515	0.9992846	0.9771759
Caxiuanã – PA/Belém – PA	**0.0249504**	**0.0038795**	0.4848264	**0.0000001**	0.9999994	0.9999996	0.5275979
Farias Brito – CE/Belém – PA	**0.0000001**	**0.0010634**	0.4848265	**0.0000001**	0.6202017	0.7837877	0.8216977
Macapá – AP/Belém – PA	**0.0001583**	0.3807651	0.4848266	**0.0008727**	0.5427934	**0.0206613**	0.5036651
Farias Brito – CE/ Caxiuanã – PA	0.9229221	0.8976012	0.4848267	0.2677705	0.8557899	0.9656668	0.1034108
Macapá – AP/ Caxiuanã – PA	0.9999988	0.1809736	0.4848268	**0.0143002**	0.7974746	0.1836252	0.9969332
Macapá – AP/ Farias Brito – CE	0.7895585	0.3410808	0.4848269	0.4184291	0.9999010	**0.0000866**	**0.0285336**

Significant values are in bold.

a From the anterior end.

**Figure 3.**
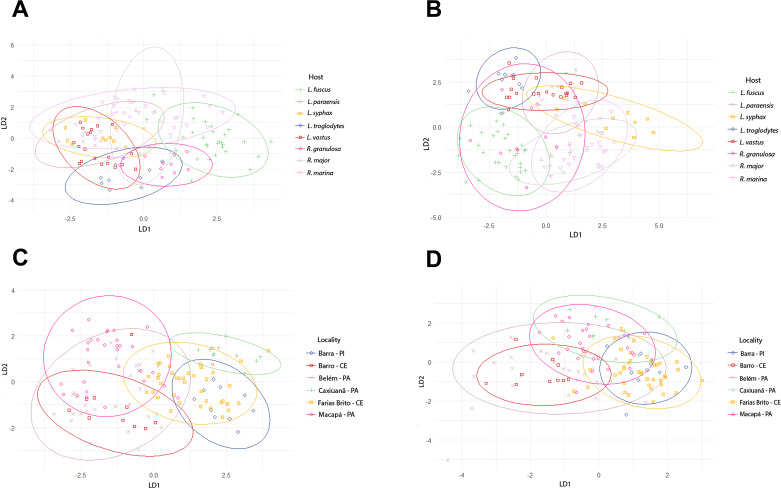
Graphs of the linear discriminant analysis of 130 female specimens and 130 male specimens of *Aplectana membranosa* from 8 hosts and 6 different localities. (A) Linear discriminant analysis graph of female *A. Membranosa* from 8 different host species, the first 2 axes account for 73% of the total observed variation; (B) Linear discriminant analysis graph of male *A. Membranosa* from 8 different host species, the first 2 axes account for 82.03% of the total observed variation; (C) Linear discriminant analysis graph of female *A. Membranosa* from 6 different localities, both axes account for 80.02% of the total observed variation; (D) Linear discriminant analysis graph of male *A. Membranosa* from 8 different host species, both axes account for 71.07% of the total observed variation. The ellipses represent the 95% confidence interval.

The results obtained by linear discriminant analysis of *A. membranosa* females by host species showed overlap between the specimens collected from all the analysed hosts, with 2 distinct *L. fuscus* groupings (Figure 3A). For the males of *A. membranosa*, the specimens collected from *L. fuscus* also formed a distinct group with less overlap compared to the other hosts (Figure 3B).

Linear discriminant analysis of locality, a variable that affected the morphometry of males and females of *A. membranosa*, revealed a group of specimens collected in the Caxiuanã National Forest in relation to female (Figure 3C); however, it was not possible to observe the same standard in males (Figure 3D).

### Molecular analysis and phylogenetics

We obtained 3 *A. membranosa* sequences from the 28S region of the ribosomal gene from specimens from 3 different locations (Belém, PA = 696 base pairs; Picos, PI, 740 base pairs; Brasilândia, Mato Grosso do Sul = 642 base pairs). We aligned our sequences with those available on GenBank, and after cutting, they generated a matrix of 17 sequences with 586 base pairs for the ingroup and 2 for the outgroup. The *Iss* index indicated no saturation in the transitions or transversions; the *Iss*.c values were higher than the Iss values.

We also observed 2% genetic divergence between the specimens of the *A. membranosa* parasites of *S. ruber* from the state of Piauí and those of *L. latrans* from the state of Mato Grosso do Sul, as well as we obtained the same value between the specimens found in *S. ruber* and *R. marina*. Among the specimens found in *L. latrans* and *R. marina*, the divergence was 1% for the same gene (Supplementary Table 1).

Our search for similar sequences from the same genomic region deposited in GenBank revealed 3 sequences from the genus *Aplectana*, 8 from the genus *Cosmocerca* and 3 from the genus *Cosmocercoides*. For the outgroup, the species chosen were *Falcaustra sinensis* and *Falcaustra* sp. (Table 2).

**Figure 4.**
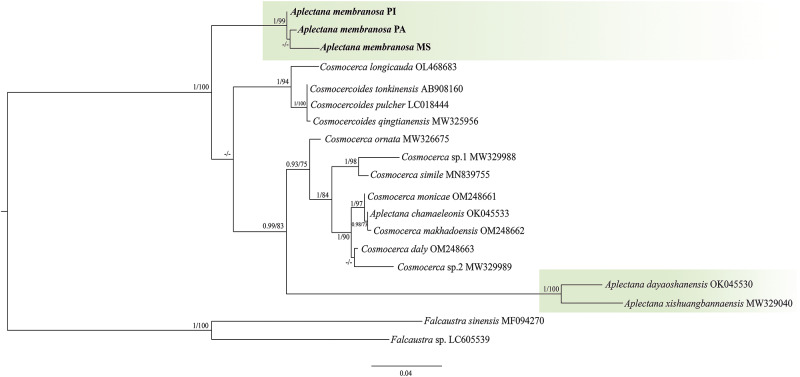
ML phylogenetic topology based on 28S sequence data using *Falcaustra* sp. and *Falcaustra sinensis* as outgroup indicating the position of *A. Membranosa* and the phylogenetic relationships of the representatives of the cosmocercidae. Support values are above or below nodes: bootstrap scores <70% are not shown or are represented by a dash. Branch-length scale bar indicates the number of substitutions per site.

The phylogenetic analyses performed using ML and BI, based on 17 taxa, showed similar topologies. We observed the formation of 2 main clades well-supported by bootstrap and posterior probability values. The phylogenetic reconstructions showed the *A. membranosa* sequences as a sister group of a larger clade, formed by 2 smaller groups: one that included sequences from *Cosmocercoides* spp. + *Cosmocerca longicauda*, and another composed of sequences from *Cosmocerca* spp. + *Aplectana* spp. (Figure 4).

We obtained 2 sequences from the *ITS1* gene from specimens from 2 locations (Belém, PA = 607 base pairs; Barras, PI = 577 base pairs). The alignment of our sequences with those available in GenBank and the cut to fit them generated a matrix of 455 base pairs with 16 sequences for the ingroup and 2 for the outgroup. The *Iss* index indicated no saturation in the transitions or transversions; the *Iss*.c values were higher than the Iss values.

For ITS1, we obtained only sequences from the state of Pará from specimens found in *R. marina* and *S. ruber* from Piauí, separated by a genetic distance of 3% (Supplementary Table 2). Our search for similar sequences from the same genomic region deposited in GenBank revealed 5 sequences from the genus *Aplectana*, 7 from the genus *Cosmocerca* and 4 from the genus *Cosmocercoides*. For the outgroup, the species chosen were *Falcaustra sinensis* and *Falcaustra* sp. (Table 2).

**Figure 5.**
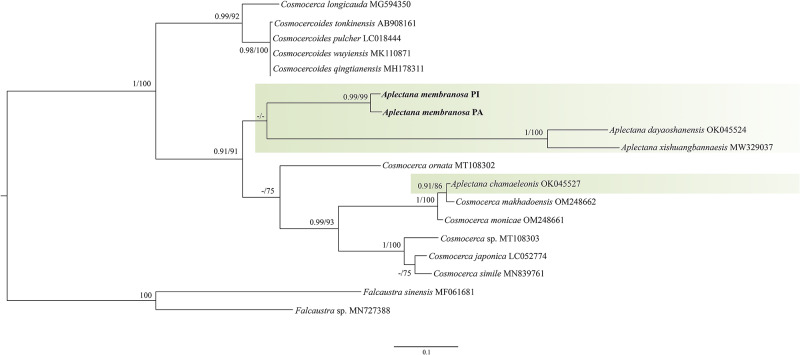
ML phylogenetic topology based on ITS1 sequence data using *Falcaustra* sp. and *Falcaustra sinensis* as outgroup indicating the position of *A. Membranosa* and the phylogenetic relationships of the representatives of the cosmocercidae. Support values are above or below nodes: bootstrap scores <70% are not shown or are represented by a dash. Branch-length scale bar indicates the number of substitutions per site.

The phylogenetic analyses performed using ML and BI, based on 18 taxa, showed similar topologies, revealing 2 main well-resolved clades by bootstrap and posterior probability values. The *A. membranosa* sequences formed a low-support clade with 2 *Aplectana* sequences (*A. dayaoshanensis* and *A. xishuangbannaensis).* The clade composed of *A. membranosa + A. dayaoshanensis*, and *A. xishuangbannaensis* was identified as a sister group of a clade that was subdivided into a branch containing a sequence of *C. ornata* and a clade that included sequences of *Cosmocerca* spp. + *A. chamaeleonis*. The sequences of *Cosmocercoides* spp. grouped into a separate clade from the others, with a branch that included a *C. longicauda* sequence (Figure 5).

### Host and occurrence records of A. membranosa

**Figure 6.**
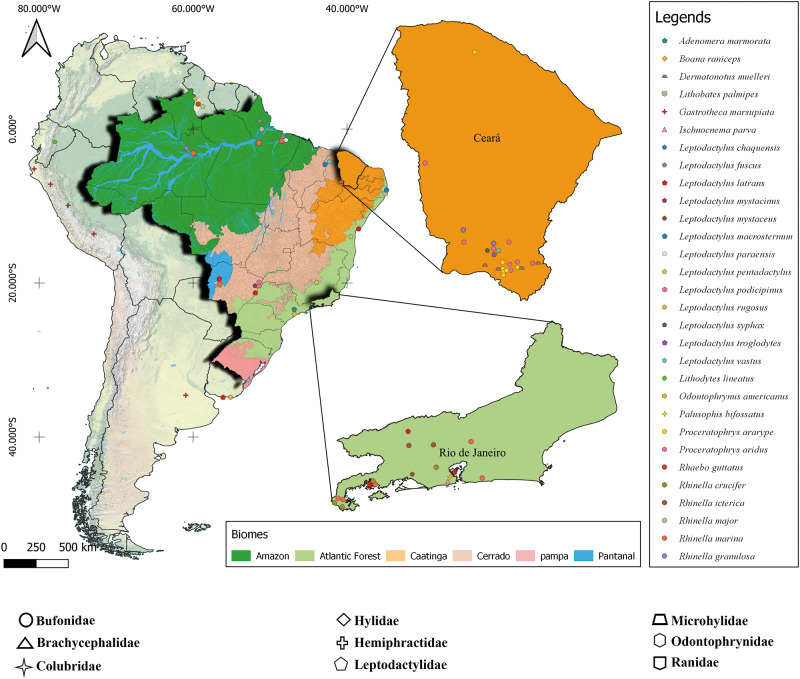
The distribution map and host species of *A. membranosa* in South America highlight the Brazilian biomes.

We found records of *A. membranosa* in 6 South American countries, Argentina, Brazil, Guyana, Ecuador, Peru and Uruguay, occurring in 8 anuran families, plus 1 record in 1 snake family. The highest occurrence reports were in Brazil, in the states of Ceará and Rio de Janeiro, covering the Caatinga and Atlantic Forest biomes, respectively. Most of the records were of amphibians of the Leptodactylidae family (Figure 6).

The host families of *A. membranosa* in Brazil included Bufonidae (6 species), Brachycephalidae (1 species), Hemiphractidae (1 species), Hylidae (1 species), Leptodactylidae (15 species), Microhylidae (1 species), Odontophrynidae (2 species), Ranidae (1 species) and Colubridae (Snake) (1 species). They occurred in 5 Brazilian biomes: Amazon, with records in the genera *Rhinella* (Bufonidae) and *Leptodactylus* (Leptodactylidae) in the states of Amazonas, Pará and Amapá; Caatinga, with records in the genera *Dermatonotus* (Microhylidae), *Leptodactylus, Proceratophrys* (Odontophrynidae) and *Rhinella* in the states of Piauí and Ceará; Cerrado, recorded only in *Leptodactylus* spp. in the states of Mato Grosso do Sul and Piauí; in the Atlantic Forest, recorded in *Leptodactylus* spp., *Boana* (Hylidae), *Ischnocnema* (Brachycephalidae) and *Palusophis* (Colubridae) in the states of Bahia, Minas Gerais, Recife, Rio de Janeiro and São Paulo; and the Pantanal, with records in the genus *Leptodactylus* in Mato Grosso do Sul (Figure 6).

## Discussion

### Taxonomic history and host-type designation

Schneider (1866) when describing *A. membranosa* presented some general characteristics common to several nematodes, such as a mouth with lips, without mentioning the number of lips or their arrangement, a posterior region of the bulb that contained a valvular apparatus and a large excretory pore located in front of the bulb. Schneider (1866) also described the vulva positioned before the anus but did not report the distance, nor did he report the morphology of the vulvar lips. Schneider (1866) also described the males with a ventrally curved tail, and the pattern of the caudal papillae in the ventral region as follows: 1 postcloacal papilla and 2–4 pairs of precloacal papillae. Furthermore, there was no additional description of the spicules, a characteristic considered of extreme importance for species identification.

In 1924, the species was redescribed by Miranda (1924), who recorded this nematode in *Leptodactylus latrans* (= *L. ocellatus*) in Manguinhos, Rio de Janeiro, Brazil. In addition to relocating the species to the genus *Aplectana*, the author added morphological and morphometric characteristics to the taxon. Miranda described the pattern of caudal papillae of males in detail, describing 5 pairs of precloacal, 2 adcloacal and 4 postcloacal papillae. However, Miranda did not clearly indicate the pairs of ad-cloacal papillae of the species, and in the redescription just-inserted 1 question mark, whereas in the illustration he represented only 1 pair. Miranda (1924) described the presence of spicules with bifurcated ends and gubernaculum.

In our study, we observed that the papillae of the specimens deposited at CHIOC (Miranda, 1924) and the specimens collected from different hosts showed the same pattern as those described in the present study. We observed that the spicules are covered by a cup-like-shaped hyaline membrane, with a concave curvature, located at the posterior end, resulting in a bifurcated appearance when observed in a lateral position (Figures 1F, G; 2D, E).

Miranda (1924) described the vulva as having 2 papillae, one on the upper lip and another on the lower lip. However, we observed in the specimens of our study and the specimens deposited at CHIOC (CHIOC 1593 and CHIOC 1594) that these structures, referred to as papillae, are cuticular protuberances similar to those of the species *A. hylambatis*. Although we did not observe the same variations in the number and size of mamelons as in *A. hylambatis* (Gonzalez et al., 2019), we emphasize that in the specimens of our study, these structures varied in size but not in quantity or position.

Travassos (1931) studied specimens of *A. membranosa* and parasites of *L. latrans* and *R. marina* without stating the exact location, reporting only as ‘Brazil’. Fahel (1952) studied *A. membranosa* of *L. latrans* and *L. pentadactylus* from Rio de Janeiro. Gonçalves et al. (2002) analysed material of *A. membranosa* parasites of *R. marina* and *R. granulosa* from Manaus, Amazonas deposited in the Helminthological Collection of the Instituto Oswaldo Cruz. In these studies, the authors presented morphometric data with some morphometric variations. Still, they upheld the pattern of caudal papillae described by Miranda (1924). However, they describe 2 pairs of adcloacal papillae (except that Gonçalves et al. (2002) did not report the pattern of caudal papillae).

In the literature review of our study, we found 8 distinct families of frogs and reptiles (Bufonidae, Brachycephalidae, Hylidae, Leptodactylidae, Microhylidae, Odontophrynidae, Ranidae and Colubridae) as hosts of *A. membranosa* in several locations in Brazil. These records (Rodrigues et al., 1982; Gonçalves et al., 2002; Luque et al., 2005; Martins and Fabio, 2005; Luque et al., 2005; Alcantara et al., 2018; Silva et al., 2019; Vieira et al., 2021; Sani et al., 2021; Mascarenhas et al., 2021; Cardoso et al., 2021; CHIOC-FIOCRUZ, 2024) corroborate the wide distribution and low host specificity of *A. membranosa*, reinforcing that this taxon is a generalist.

### Morphological and morphometric variation

In the 260 specimens of *A. membranosa* analysed in our study, there was no morphological variation in the spicules, gubernaculum in males or vulvar protuberances in females. Regarding the morphology of the spicules, all males presented spicules with the presence of a hyaline membrane. The membrane is being presented for the first time in this study; previously, other authors only described the spicule as ‘having a bifurcated’ aspect (Schneider, 1866; Miranda, 1924).

One of the possible explanations for the absence of a description of the membrane by several authors (Miranda, 1924; Travassos, 1931; Gonçalves et al., 2002) may be that the structure is delicate and difficult to visualize and may even collapse in the processes necessary for examination. Regarding the morphology of the vulva in females, all specimens showed 2 protuberances on the lips of the vulva (1 on each lip). These results differ from those found by González et al. (2019) in a similar study of *A. hylambatis*: They found specimens with morphological differences by host and location, especially in the spicules, gubernaculum and vulvar protuberance.

Here, we observed only morphometric variations between the *A. membranosa* specimens of the hosts and localities studied, such as distance from the excretory pore to the anterior end, distance from the vulva to the anterior end, spicule size and gubernaculum size, as well as morphometric variation compared to previous studies (Table 3). These data highlight the wide variation in these traits, especially concerning different hosts. We found specimens of *A. membranosa* from hosts with a small body size (for example, *L. troglodytes*) are usually smaller than those obtained from larger body size hosts such as *L. latrans/R. marina* (Table 3).

Similar results were found by Sukee et al. (2018) in *Pharyngostrongylus kappa* Mawson, 1965, and Rhoden and Bolek (2011) in *Gyrinicola batrachiensis* (Walton, 1929), considering the morphology, life cycle and ecology of *Gyrinicola batrachiensis*. Thus, we emphasize how the biology of this helminth species relates to different hosts and habitats, without considering the phylogenetic context addressed by Walker et al. (2024). These authors found significant morphological and morphometric variation among the analysed *Gyrinicola* specimens, consistent with genetic tests indicating the presence of distinct species. They highlighted the role of the host concerning habitat and geographic distribution, as well as the geographic barriers evidenced. This pattern of coevolution, driven by ecological specialization and geographic isolation, promoted the diversification observed in their study, resulting in genetically and morphologically distinct lineages. In contrast, our study did not observe morphological differences among the analysed specimens of *Aplectana membranosa*, and the few identified morphometric variations are not considered interspecific.

We observed that the morphometric characters corpus length, length and width of the bulb, distance from the nerve ring to the anterior end, the distance between the excretory pore and the anterior end, tail length, the distance from the vulva to the posterior end, and the length and width of the eggs of females of *A. membranosa* were the main factors affecting the observed variability (Table 4).

Regarding males of *A. membranosa*, we found that the total body length, the length of the oesophagus, the length of the corpus, the distance from the excretory pore to the anterior end, the tail length relative to the posterior end, the size of the gubernaculum and the size of the spicule are the factors that most influence morphometric variation (Table 5). Among these characters, the size of the spicules is one of the main characteristics used in the identification of *Aplectana* species, because it is a character used to calculate the proportion relative to body length (Walton, 1940; Silva, 1954; Baker, 1980; Baker and Vaucher, 1986; Ramallo et al., 2008; Falcón-Ordaz et al., 2014; Piñeiro-Gomez et al., 2017; González et al., 2019). However, in the present study, we observed morphometric variation in this trait according to the size of the host (Table 2), as observed in previous studies (see Fahel, 1952; Gonçalves et al., 2002). These data indicate a considerable variation in this trait, and we suggest that the size of the spicules should not be used to identify *A. membranosa*.

In the present study, all 9 morphological characters highlighted by the PCA in females and the 5 highlighted characters in males showed statistically significant differences between hosts and localities (Tables 6 and 7). In the study by González et al. (2019), females of *A. hylambatis* showed significant differences in all morphological and morphometric characters besides the distance from the vulva to the posterior end and total body length.

Comparisons between different species hosts showed that all females and all males of *A. membranosa* differed in at least 1 metric characteristic, except for females of some host pairs of congeneric species, such as *L. vastus/L. troglodytes, R. major/R. granulosa* and *R. marina/R. major* and some pairs of host species from different families such as the case of *R. major/L. fuscus, R. major/L. paraensis, R. major/L. syphax, R. granulosa*/*L. troglodytes, R. major/L. R. granulosa*/*L. troglodytes* and *R. major/L. vastus* (Table 8). We observed a high degree of dissimilarity for males, in some cases involving species of different genera and congeneric species, such as *L. troglodytes* and *L. syphax* (Table 9); even though they belong to the same family, we believe that the dissimilarity observed in this case may reflect the influence of the individual’s body size, but it was not tested in this study. Our results also corroborate the findings of other authors (Rodrigues et al., 2004; López et al., 2009; Solé and Rödder, 2010; González et al., 2019) who observed morphological and morphometric variation associated with hosts species.

The degree of dissimilarity between pairs of species of different genera can be explained by the position and phylogenetic relationship of the hosts, as mentioned by González et al. (2019), with specimens of *A. hylambatis* collected from hosts of different families (bufonids, leptodactylids and hylids). This character also reflects the amphibians’ physiological and behavioural differences, emphasizing what Kirillov and Kirillova (2015) observed in their evaluation of the variability and determining factors of the size structure of *Cosmocerca ornata*. The authors concluded that the greater the differences in the biology and ecology of the hosts were, the greater the variability in the body size of *C. ornata.*

Regarding locality, males and females of *A. membranosa* showed significant differences in morphometric measurements between all collection sites (Tables 10 and 11). González et al. (2019) reported morphometric variation in *A. hylambatis* between individuals collected in 7 different locations in Argentina, and Vhora and Bolek (2013) reported morphometric variation in *A. hamatospicula* from Oklahoma when comparing the measurements with previous records of specimens collected in Mexico and Cuba.

We observed that females of *A. membranosa* showed more significant dissimilarity between individuals collected in the National Forest (FLONA) of Caxiuanã, PA, and those collected in the municipality of Barro, CE. This result may be related to the ecological conditions of both localities since the FLONA Caxiuanã-PA is located within the Amazon forest, with a humid equatorial climate. Barro, CE is in the Caatinga biome, with a predominantly semiarid climate, reinforcing the hypothesis that environmental conditions such as temperature and latitude can influence the size of parasitic helminths (Dallas et al., 2019). However, genetic divergence studies of *A. membranosa* specimens from both localities are necessary to corroborate this hypothesis, which we were unable to achieve in our study.

The linear discriminant analysis graphs (Figure 3A–D) compare females and males of *A. membranosa* collected from different hosts and locations. They show that females of *A. membranosa* collected from *L. fuscus* were grouped separately from those isolated from the other hosts, forming a distinct grouping (Figure 3A). The same occurred for the males collected from *L. fuscus* (Figure 3B).

The host species *L. fuscus* was the only 1 collected in 3 locations that belong to different states, namely, Belém, PA, Macapá, AP and Barro, CE, representing different microhabitats. By location, the females overlapped in the linear discriminant analysis graph (Figure 3C), highlighting the similarity between the specimens collected from various regions. The males of *A. membranosa* collected at the sampled locations showed the same groups observed for females. Unlike the other areas, the Caxiuanã FLONA is characterized as an insular federal conservation area of the Marajó archipelago, where tropical humid *terra firme* forest is the predominant vegetation (Lisboa et al., 1997), yielding environmental and ecological conditions that are different from those in other locations that may be strongly influenced by anthropization.

The males of *A. membranosa* from different locations showed a more significant dissimilarity in 2 collecting sites in the same state, Farias Brito and Barro in Ceará, probably because the largest number of different host species were collected in both locations, including *R. granulosa, L. vastus, L. troglodytes, L. syphax* and *L. fuscus*. However, the discriminant analysis plot generally shows the *A. membranosa* male specimens heavily overlapping (Figure 3D).

The results obtained from the statistical analyses suggest that species of the genus *Aplectana* are prone to metric variation induced by the host and locality. Such variations are common in amphibian parasitic nematodes (Rhoden and Bolek, 2011). Among the factors that influence these variations are age, sex, host species, number of parasites found in the host and seasonal changes (Kirillov and Kirillova, 2015; Vakker, 2018; González et al., 2019; Tarasovskaya and Zhumadilov, 2019; Kirillova et al., 2021).

### Genetic divergence and phylogenetic analysis

This study presents the first insights into the genetic divergence between specimens of *A. membranosa* from different hosts and geographic regions, as well as the first phylogenetic study of this species, corroborating that the genus *Aplectana* is paraphyletic, as observed in previous studies (see Tran et al., 2015; Chen et al., 2021b; Svitin et al., 2023).

We observed a 2% nucleotide divergence in the *28S rRNA* gene between the sequences of the *A. membranosa* parasites *S. ruber* from the State of Piauí and *L. latrans* from the State of Mato Grosso do Sul and those found in *R. marina* of the State of Pará. In contrast, the divergence of the *28S* gene between the specimens parasitizing *L. latrans* and *R. marina* in the states of Pará and Piauí was 1%, indicating high intraspecific variation.

Although the *28S* gene is widely recognized as highly conserved, it consists of a combination of conserved and divergent regions, referred to as ‘divergence regions – D’ (Hassouna et al., 1984). This combination of conserved and divergent regions results in nucleotide variations in the gene, which can indicate genetic separation between different groups of individuals of the same species, especially in allopatric contexts (Sonnenberg et al., 2007), where populations are geographically isolated, as observed in the present study. Over time, this process can lead to adaptations to specific environments, promoting changes in genetic sequences. Additionally, the use of ribosomal genes may present some challenges, such as the presence of pseudogenes and intragenomic variation (Sonnenberg et al., 2007), which can make the interpretation and integrity of genetic data difficult.

Significant genetic divergence among specimens from different regions and hosts reflects the possibility of adaptation to specific environments, as observed with *R. marina* and *L. latrans* (both terrestrial habitats) and *S. ruber* (arboreal habitat). This point was also addressed by Walker et al. (2024) in their study, where they considered phylogenetic patterns and genetic divergence in *Gyrinicola* and the relationship to the aquatic or semi-aquatic habitats of their hosts. Walker et al. (2024) discussed environmental adaptation and how these adaptations can be reflected in phylogenetic relationships. This aligns with what we found in the present study on *Aplectana membranosa*, where the observed genetic divergence suggests that environmental factors may have influenced genetic separation and diversity within the species, leading to differences in genetic sequences among hosts with distinct habitats.

For the ITS1 region, the 3% genetic divergence between the *R. marina* specimen from the state of Pará and the *S. ruber* specimen from the state of Piauí is considered high. However, when compared to the variability observed in members of the family Cosmocercidae, which exhibit high genetic variability overall (genetic divergence range among *Aplectana* spp. 15–45% and among *Cosmocerca* spp. 4–39%), this variation can be not representing an interspecific.

Although the species of *Cosmocercoides* (*Cosmocercoides qingtianensis, Cosmocercoides pulcher, Cosmocercoides tonkinensis* and *Cosmocercoides wuyiensis*) are considered valid, the sequences deposited in GenBank for 28S and ITS1 showed 0% genetic divergence in our analyses, which contrasts with the species of other genera in the family Cosmocercidae. Therefore, we cannot consider them for comparison, due to the absence of type specimens or vouchers for certain *Cosmocercoides* species in GenBank, this posed a significant challenge, limiting the inclusion of these species in phylogenetic analyses. Such a limitation compromises the representation of genetic diversity and evolutionary relationships within the group. Furthermore, according to the original descriptions, these species also show few morphological differences (Wilkie, 1930; Tran et al., 2015; Chen et al., 2018; Liu et al., 2019). Additionally, the variations in genetic divergence found in our study differed from those of Chen et al. (2021a), who did not identify any genetic divergence between specimens of *A. xishuangbannaensis* at the ITS1 or 28S region.

Despite the high genetic divergence between the *A. membranosa* specimens in our study, the results indicate a relationship between the parasites of hosts with similar (terrestrial) habitats, such as *R. marina* and *L. latrans*. In contrast, the parasitic specimens of *S. ruber*, which has an arboreal habit, showed greater genetic distance than the other specimens.

We observed that *Cosmocerca* had a closer phylogenetic relationship to *Aplectana* spp. The phylogenies recovered in the present study demonstrated that *A. chamaeleonis* is a sister species of *Cosmocerca makhadoensis*, showing that it is phylogenetically distant from its congeners and closer to *Cosmocerca* spp.

As previously suggested, the phylogenetic position of *A. chamaeleonis* may reinforce the paraphyly of *Aplectana*, or the species may be mistakenly identified. Notably, studies in which genetic data on this species were provided lack morphological information that would allow confirmation of that species’ identity (see Sinsch et al., 2020; Chen et al., 2021b; Andrus et al., 2022). Thus, we hypothesized that the sequence belongs to the genus *Cosmocerca*.

Regarding the phylogenetic position of *Cosmocerca longicauda*, in the study conducted by Sinsch et al. (2018), the sequence we used for the analyses with the *28S* gene is presented with a low-resolution photomicrograph of the male tail of *C. longicauda*. Despite the image’s limited quality, we compared the morphology of the gubernaculum and spicule of *C. longicauda*, as described by Travassos (1931) and Sinsch et al. (2018). We observed that the morphology of the gubernaculum and spicule are different in the studies. For example, Travassos (1931) characterized the gubernaculum as well-sclerotized and longer than the spicules; moreover, the papillae with plectanes are pretty evident. In the study by Sinsch et al. (2018), the spicules are longer than the gubernaculum, which is less sclerotized, and it is not possible to observe papillae with plectanes, a generic characteristic of *Cosmocerca*.

Thus, we observed that the morphological traits of the specimens from Sinsch et al. (2018) are more similar to those found in species of the genus *Cosmocercoides*, suggesting that the gene sequence of *C. longicauda* deposited in the GenBank database belongs to the genus *Cosmocercoides*.

The sequence corresponding to the *28S* gene of *A. membranosa* reveals a distant and well-supported relationship (100%) with its congeners, positioning it as a sister group of *Cosmocercoides* spp. + *Aplectana* spp. + *Cosmocerca* spp. However, in the phylogenetic reconstruction using the *ITS1* gene, *A. membranosa* is closer to *A. dayoashanensis* + *A. xishuangbannaensis*, with low support (55%). This clustering difference between the genes highlights that the phylogenetic relationships are not yet well established and may change with the inclusion of more *Aplectana* species.

Furthermore, in our analysis of the *28S* gene, we observed that *A. membranosa* formed an independent group (Neotropical). When investigating the phylogenetic position of *Cosmocercoides amapari* Rebêlo, Santos and Melo, 2022, based on the *Cox1* gene, Rebêlo et al. (2023) also found that the species formed a clade isolated from its congeners, suggesting that this grouping reflects the geographical location of the species. Thus, our data corroborate that their biogeographic region may influence the separation of these clades from Cosmocercidae.

The *ITS1* gene is the most suitable for distinguishing species belonging to the family Cosmocercidae, so we should note that the comparative analysis between *A. dayoashanensis* + *A. xishuangbannaensis* and *A. membranosa* (considering the *ITS1* gene) revealed high genetic divergence (34% and 35%, respectively). These values are similar to the divergence between distinct genera, exemplified by the comparison between *Cosmocercoides* and *Aplectana* (35%). This result demonstrates the effects of geographic distance and may indicate that the lineage of the eastern species diverged long ago. It is also possible that *A. dayoashanensis* and *A. xishuangbannaensis* represent a genus that has not yet differentiated morphologically from *Aplectana*.

The genetic variation observed for the ITS1 of *A. membranosa* is intraspecific and host-related, but this variation may indicate the beginning of interspecific differentiation. According to Rahmouni et al. (2020), a host lineage’s ecology can influence its parasite community’s speciation potential. *A. membranosa* is a generalist species found in frogs of different host lineages and sizes that explore different habitats. Such characteristics favour an increase in gene flow and make the species susceptible to this process of interspecific differentiation.

The limited number of deposited sequences of specimens from specific geographic regions or hosts may introduce significant bias, hindering the assessment of genetic diversity and phylogenetic relationships. This limitation can result in an inaccurate representation of the variability within populations of *Cosmocercoides, Cosmocerca*, and other species of the genus *Aplectana*.

The substantial genetic variability observed among helminths of the family Cosmocercidae, with divergences ranging from 15% to 45% between species, highlights the complexity and extent of genetic diversity, even when using ribosomal genes such as *28S rRNA* and *ITS1*. This reflects a long history of adaptation and speciation. However, challenges such as the presence of pseudogenes and intragenomic variation may difficult data interpretation.

The absence of representative sequences for all species further limits comprehensive analyses of genetic divergence and phylogenetic relationships, creating gaps in the understanding of their evolution and diversification. Thus, future studies employing molecular tests for species delimitation, complemented by morphometric analyses, are essential to determine whether *Aplectana membranosa* specimens represent distinct species.

### Final remarks

This study obtained the first sequences of the *28S rRNA* gene and the ITS1 region of *A. membranosa* to be deposited in GenBank, made the first examination of the morphological and morphometric variation of the taxon and is the first to determine the distribution of the taxon in South America.

Furthermore, with the aid of scanning electron microscopy, we presented the spicules of *A. membranosa* in more detail, adding the presence of a bifurcated hyaline membrane – cup-like shape, and reviewed the number and arrangement of the caudal papillae, which have been presented differently by different authors (see Schneider, 1866; Travassos, 1931; Fahel, 1952), reinforcing the representation of the papillae represented in light microscopy by Miranda (1924), as 1 ad-cloacal pair + 4 pairs postcloacal, with the remaining papillae being distributed as described in the literature (5 pairs precloacal; 3 pairs in the upper lip of the cloaca). We did not find numerical variation by host or location in Brazil. Thus, the specimens in our study resemble to those described by Miranda (1924). Therefore, we designate that Miranda’s specimens should represent *A. membranosa*, and the vouchers deposited in the Oswaldo Cruz Helminthological Collection are the neotypes of the species.

Regarding the spicules and gubernaculum, we found no variation in morphology by host or locality. Males of *A. membranosa* have 2 long, subequal spicules covered with a hyaline membrane, which has a spatulate morphology at the distal end. The gubernaculum is concave and well sclerotized. We found no difference in vulvar morphology between females; however, we emphasize the existence of 2 protuberances on the vulvar lips.

Furthermore, through statistical tests, we show that males and females of the species exhibit significant variability in morphological measurements, taking into account the host and locality, especially the variation in the length of the spicules and gubernaculum in males, as indicated by previous studies of species of the genus *Aplectana* that possess both characters as essential morphological characteristics for the identification of the helminths of this group.

In general, the metric characters of this cosmocercid vary depending on whether the host or the locality in which the host lives is considered, including characters deemed relevant to the description of the taxon. It is important to note that the *A. membranosa* nematodes found in *L. fuscus* form a differentiated group compared to the others, as visualized by the linear discriminant analysis graph. We can attribute this to the fact that the host species *L. fuscus* was the only 1 collected in 3 localities in different states and representing different microhabitats, reinforcing the hypothesis that seasonal differences, temperature and geographic characteristics are related to factors influencing the observed metric variations.

Molecular analysis revealed that ITS1 is an excellent molecular marker for the differentiation and identification of Cosmocercidae; however, the *28S* gene provides new interpretations of the evolutionary history of the family Cosmocercidae and leaves questions to be answered that could help us better understand the phylogenetic relationships of the family Cosmocercidae, such as: What happened evolutionarily for the *Aplectana* species to diverge from each other? Could *A. dayoashanensis* and *A. xishuangbannaensis* represent a genus that has not yet been morphologically differentiated from *Aplectana*? Therefore, conducting a more robust sampling to investigate these issues is still necessary. Additionally, these future studies will require more sequences of species of Cosmocercidae provided from vouchers/hologenophores, to confirm the morphological identification of the taxon.

Through genetic data, we determined the relationships of *A. membranosa* within Cosmocercidae, confirming that their separation is related to geographic distribution, which we observed through the analyses of the 2 genes. However, obtaining sequences from specimens from all the studied locations was difficult, which hindered the complete analysis of the family and contributed to the lack of data on Cosmocercidae in the genetic databases.

The results of this study reiterate the importance of using morphological, morphometric and molecular data so that the taxonomic and evolutionary history of the groups of nematodes concerning their hosts can be better elucidated. Our study represents an advance in research encompassing morphological variations within the genus *Aplectana* and associated factors. More studies using integrative approaches are needed to fill the gaps in the molecular data available for the Cosmocercidae family.

We emphasize the need for a prior morphological analyses of any specimens studied by molecular-biological methods, especially when the goal is not to provide species descriptions; there must be a deposit of parasite testimonies because evidence of the presence of the parasites in space and time must be available to the scientific community through well-curated collections. Such practices will be essential for obtaining more accurate data, favouring future systematic studies and taxonomic delineation of the family Cosmocercidae.

## Supporting information

Santos et al. supplementary material 1 Santos et al. supplementary material

Santos et al. supplementary material 2 Santos et al. supplementary material

## Supplementary material

The supplementary material for this article can be found at https://doi.org/10.1017/S0031182025000204.
